# MOICS, a novel classier deciphering immune heterogeneity and aid precise management of clear cell renal cell carcinoma at multiomics level

**DOI:** 10.1080/15384047.2024.2345977

**Published:** 2024-04-24

**Authors:** Ying Liu, Lin Qi, Bicheng Ye, Anbang Wang, Juan Lu, Le Qu, Peng Luo, Linhui Wang, Aimin Jiang

**Affiliations:** a Department of Urology, Changhai Hospital, Naval Medical University (Second Military Medical University), Shanghai, China; b Department of Orthopedics, The Second Xiangya Hospital, Central South University, Changsha, Hunan, China; c Hunan Key Laboratory of Tumor Models and Individualized Medicine, The Second Xiangya Hospital, Changsha, Hunan, China; d School of Clinical Medicine, Medical College of Yangzhou Polytechnic College, Yangzhou, China; e Department of Urology, Changzheng Hospital, Naval Medical University (Second Military Medical University), Shanghai, China; f Vocational Education Center, Naval Medical University (Second Military Medical University), Shanghai, China; g Department of Urology, Affiliated Jinling Hospital, Medical School of Nanjing University, Nanjing, Jiangsu, China; h Department of Oncology, Zhujiang Hospital, Southern Medical University, Guangzhou, China

**Keywords:** Clear cell renal cell carcinoma, immune phenotype, multiomics, drug sensitivity, prognostic biomarker

## Abstract

Recent studies have indicated that the tumor immune microenvironment plays a pivotal role in the initiation and progression of clear cell renal cell carcinoma (ccRCC). However, the characteristics and heterogeneity of tumor immunity in ccRCC, particularly at the multiomics level, remain poorly understood. We analyzed immune multiomics datasets to perform a consensus cluster analysis and validate the clustering results across multiple internal and external ccRCC datasets; and identified two distinctive immune phenotypes of ccRCC, which we named multiomics immune-based cancer subtype 1 (MOICS1) and subtype 2 (MOICS2). The former, MOICS1, is characterized by an immune-hot phenotype with poor clinical outcomes, marked by significant proliferation of CD4+ and CD8+ T cells, fibroblasts, and high levels of immune inhibitory signatures; the latter, MOICS2, exhibits an immune-cold phenotype with favorable clinical characteristics, characterized by robust immune activity and high infiltration of endothelial cells and immune stimulatory signatures. Besides, a significant negative correlation between immune infiltration and angiogenesis were identified. We further explored the mechanisms underlying these differences, revealing that negatively regulated endopeptidase activity, activated cornification, and neutrophil degranulation may promote an immune-deficient phenotype, whereas enhanced monocyte recruitment could ameliorate this deficiency. Additionally, significant differences were observed in the genomic landscapes between the subtypes: MOICS1 exhibited mutations in TTN, BAP1, SETD2, MTOR, MUC16, CSMD3, and AKAP9, while MOICS2 was characterized by notable alterations in the TGF-β pathway. Overall, our work demonstrates that multi-immune omics remodeling analysis enhances the understanding of the immune heterogeneity in ccRCC and supports precise patient management.

## Introduction

Renal cell carcinoma (RCC) is one of the most common and lethal cancers in the urogenital system. Over the past decades, the incidence and mortality of RCC have been increasing worldwide. RCC contains various pathological types. Among them, clear cell renal cell carcinoma (ccRCC) is the most common type of RCC, accounting for nearly 80% of RCC cases.^1^ Recent decades have witnessed the advancement of comprehensive therapeutic strategies in ccRCC, among which targeted and immune therapies have significantly prolonged the overall survival time of patients. Unfortunately, the heterogeneity of ccRCC determines the distinctive clinical outcome and notorious therapy resistance.^2,3^ Recently, immunotherapy has become the cornerstone of therapies for advanced ccRCC patients. However, ccRCC patients face intolerable adverse events and limited clinical benefit, to some extent, which could be explained by the intra- and intertumoral heterogeneity and dynamic reprogramming of the tumor immunological microenvironment (TIME).^4,5^ Current risk stratification systems, including the TNM and AJCC staging systems, are widely used in clinical practice but fail to precisely manage ccRCC patients.^6^ Since ccRCC has a very high degree of genetic heterogeneity between tumors,^7^ patients with the same TNM stage could have different prognoses despite receiving the same standardized treatment, and it is urgent to develop a more precise and robust prognostic stratification system. Recently, molecular classification has allowed for better prediction of prognosis and has provided abundant information for treatment decision-making.^8^ Owing to tremendous advancements in omics sequencing and integration algorithms, it is promising to conduct a multi-immune omics remodeling system to guide the personalized management of advanced ccRCC patients.

The tumor microenvironment is a highly dynamic and complex system affecting tumor occurrence, development and metastasis.^9^ Accumulating evidence indicates that the TIME determines immune evasion and sensitivity to immune checkpoint block therapy.^10,11^ It is worth noting that ccRCC is one of the tumor types with the highest T-cell infiltration level.^12,13^ Due to the great success in cancer immunotherapy, immune checkpoint inhibitors (ICIs) are recommended as the first-line treatment for ccRCC.^14^ However, patients with ccRCC experience minimal or no clinical benefit from immunotherapy because of tumor heterogeneity. Meanwhile, acquired drug resistance frequently develops in patients who are sensitive to immunotherapy as the initial treatment.^15^ The immune status in the tumor microenvironment and the potential mechanisms associated with immune escape in ccRCC remain unknown.

In this study, we identified and verified two distinct immune-related subtypes through multiomics analysis in ccRCC. Furthermore, we established and verified a robust five-gene-based risk model to predict the prognosis of patients with ccRCC and identified a novel prognosis-related secreted protein that could help guide precision medicine for ccRCC.

## Method and materials

### Data collection and processing

The pipeline of this work is depicted in Figure S1. Clear cell renal cell cancer normalized expression profiling data, DNA methylation data, tumor mutation burden (TMB), microsatellite instability (MSI), copy number variation (CNV) and somatic mutation data and clinical characteristics were downloaded from the UCSC XENA database (*n* = 243).^16^ External ccRCC cohort, including E-MTAB-1980 (*n* = 100), which included expression profile and prognostic information, was downloaded from the Express-array database; advanced ccRCC patient samples of therapy-naïve surgical resected from study (Accession ID: EGAS00001004290, EGAS00001004291, EGAS00001004292, *n* = 823); proteogenomic data in the ProteomeXchange Consortium (Accession ID: PXD030344, *n* = 133); and patients received immune therapy were collected from Braun’s study (*n* = 311).^17^ In addition, locally advanced or metastatic urothelial bladder cancer received atezolizumab (Accession ID: IMvigor210, *n* = 348) were unitized as the testing datasets to evaluate the accuracy of the prediction of our risk subtyping system of ICIs sensitivity. The baseline information of datasets enrolled in our work were summarized in Table S1. This study also facilitated several public cancer databases, including TIMER and TIDE database(Figure S1(a)).^18^ Patients who lack of multi omics datasets and with an OS time of less than one month was excluded to enhance the robustness of downstream analyses. The degree of variation in continuous variables (including mRNA, lncRNA, miRNA, CNA and methylation) was assessed by setting the method parameter to mad within the getElites function in MOVICS. This allowed for the selection of the top 1,500 genes showcasing the highest extent of variation. Subsequently, the prognostic significance of these genes was determined by setting the method parameter to cox and integrating clinical data. Genes were considered prognostically significant at a level of *p* < .05 across each dimension of data. For binary gene mutation data, we initially applied the oncoPrint function from the maftools package to identify the top 5,000 genes exhibiting the highest mutation rate. Ethical Review Committee approval and informed consent were not required for datasets downloaded from public datasets. Patients lacking clinical information or expression profiles were excluded from the study.

### Identification of the optimal cluster number

To calculate the optimal number of clusters k, k should be large enough to identify subgroup differences and small enough to avoid redundancy. Facilitated with R package MOVICS, which applied Gaps-statistics and Clustering Prediction Index (CPI) to determine the most robust cluster number.^19^ Then, we utilized 10 mainstream cluster algorithms, including COCA, ConsensusClustering, CLMLR, IntNMF, iClusterBayes, PINSPlus, NEMO, MoCluster, LRAcluster and SNF, to perform cluster analysis (Figure S1(b)). With the consensus ensemble principle, clustering results from the algorithms mentioned above were introduced and integrated to improve the robustness of the remodeling results. The detailed parameters and principles can be found in our previous study.^20^

### Enrichment analysis based on differentially expressed genes

The R package DEseq2 was applied to perform differentially expressed gene (DEG) analysis between subgroups, and thresholds were set at adjusted *p* < .01 and abstract log2 fold change > 2.^21^ After calculating DEGs, we performed functional analysis including GO, KEGG, GSEA and GSVA analysis via the R package ClusterProfiler to explain the molecular mechanism between MOICS1 and MOICS2 (Figure S1(c)).^22^ All gmt files were downloaded from public datasets, including MSigDB and ConsensusPathDB.^23,24^

### Differences in immune infiltration signatures and therapy response

We utilized multiple immune cell infiltration algorithms, including CIBERSORT, EPIC, MCPCOUNTER, QUANTISEQ, TIMER and XCELL, to decode the different TIMEs between MOICS1 and MOICS2 (Figure S1(c)).^18,25–27^ Employing the IOBR package, we compiled a multitude of previously documented signatures pertinent to tumor microenvironment (TME) cell types and responses to immunotherapy. Utilizing a standardized method, we calculated enrichment scores for each sample, enabling a comprehensive analysis of immunological disparities across different clusters. In addition, single-sample gene set enrichment analysis (ssGSVA) was also utilized to validate such differences.^28^ The R package ESTIMATE was used to calculate immune and tumor purity. The Tumor Immune Dysfunction and Exclusion (TIDE) algorithm was applied to illustrate the different immunotherapy sensitivities.^29^

### Mutation spectrum characteristics between subgroups

Somatic data were analyzed and visualized via the R package Maftools to compare mutational patterns between subgroups.^30^ With the aid of correlation functions in the R package Maftools, the tumor mutation panorama, base transitions and transversions, single nucleotide variants, mutation rates of alleles, copy number mutations, mutually exclusive or coexisting mutations, and gene mutation survival rates were calculated. Through the transformation analysis function module, the drug and gene interactions and the differences in oncogenic signaling pathways of different subsets were also analyzed. Analysis of recurrent extensive and focal somatic copy number alterations (CNAs) was deciphered via the GISTIC 2.0 algorithm based on the Euclidean distance of the threshold copy number (Figure S1(c)).^31,32^

### Drug susceptibility prediction

Each patient was assessed for their susceptibility to molecular drugs using the Genomics of Cancer Drug Sensitivity (GDSC) database.^33^ The R package pRRophetic was used to estimate the half-maximal inhibitory concentration (IC50) and cross-validate, and we introduced ridge regression to evaluate the accuracy of such drug sensitivity differences. In addition, we employed the compDrugsen function from the MOVICS R package to analyze drug sensitivity across a broad spectrum of chemotherapeutic and targeted drugs. This approach was chosen to explore the potential differential sensitivity of MOICS1 or MOICS2.^34^ In detail, a lower IC50 represents higher sensitivity to chemotherapy (Figure S1(c)).^35^

### Construction of a risk prediction model based on subgroup biomarkers

First, using subgroup-related biomarkers and overall prognostic information from the TCGA-KIRC cohort, we filtered the prognostic signature through univariate Cox regression analysis. Then, the Random Survival Forest Variable Hunting (RSFVH) algorithm was further performed to select crucial signatures (Figure S1(d)). Finally, a risk scoring model was constructed using the best combination of prognostic genes for screening. The JAPAN-ccRCC, IGCG-EU, and Braun’s and Motzer’s cohorts were used to validate our risk scoring model; compared with the median risk score, all patients were classified into high- and low-risk subgroups.

### Analysis of plasma proteins level of SAA4 in ccRCC patients

Peripheral blood samples of all enrolled ccRCC patients were collected in a vacuum blood collection tube containing EDTA and centrifuged at 4000 rpm for 10 min at 4°C to obtain plasma samples, which were stored at −80°C until further operation. We randomly selected plasma samples from 20 patients before and after surgery for further analysis. We verified the expression level of SAA4 via commensurable ELISA kits purchased from RayBiotech Life, Inc.

### Investigating the biological role of SAA4 in renal cancer

We utilized the strategy of guilt-of-association to investigate the biological roles of SAA4 in ccRCC. In detail, we firstly calculated the spearman’s correlations index between SAA4 and remained protein coding genes retrieved from TCGA. Sorting by the level of association between other genes and SAA4, genes most related to SAA4 expression were selected for enrichment analysis according to the guilt-of-association method (*p* < .05 and abstract cor > 0.5). ClusterProfiler package was used to perform functional annotation analysis (GO) and gene set enrichment analysis (GSEA) on the most relevant related genes. Human recombinant protein of SAA4 (HY-P71277) was purchased from MedChemExpress, which was added to cell culture medium of 786–0 and ACHN.

### Statistical analysis

R software (version 4.0.4) was used to perform plotting data processing and statistical analysis. The Kruskal‒Wallis test and Wilcoxon test were used to compare differences between subgroups. The chi-square test was utilized to investigate differences in clinical characteristics and therapy response. The Kaplan‒Meier method and log-rank test were applied to perform analysis of patient survival time. Univariate Cox regression and multiple Cox regression algorithms were introduced to calculate hazard ratio (HR) differences. All statistical tests in this study were two-sided, and *p* < .05 was considered statistically significant.

## Result

### Landscapes of the two distinctive immune subgroups

**Figure 1.**
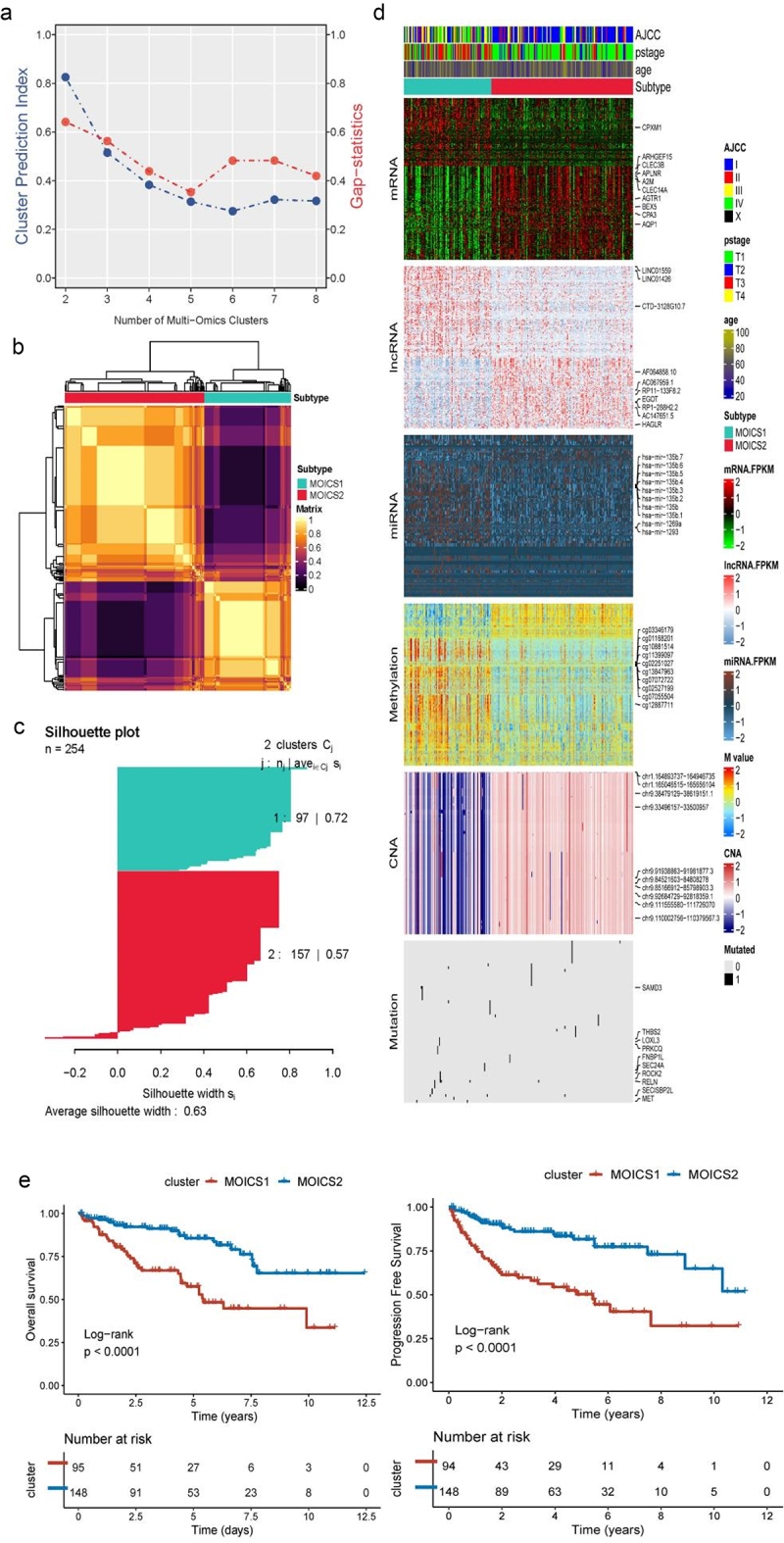
The landscape of multiomics differences between MOICS1 and MOICS2.

The optimal number of immune subgroups was estimated to be 2 with CPI and Gap statistics mentioned previously (Figure 1(a)). The consistency of the consensus cluster results is illustrated in the correlation heatmap and silhouette map in Figure 1(b,c). The two immune subtypes presented differences in multiomics landscapes (Figure 1(d)). CPXM1, ARHGEF15, CLEC3B, APLNR, A2M, CLEC14A, ARTR1, BEX5, CPA3 and AQP1 were the top mRNAs that showed significant differences between MOICS1 and MOICS2; LINC01559, LINC01426, CTD-3128G10.7, AF064858.10, AC067959.1, RP11-133F8.2, EGOT, RP1-288H2.2, AC147651.5, and HAGLR were the most differential lncRNA; has-let-135b.7, has-let-135b.6, has-mir-135b.5, has-mir-135b.4, has-mir-135b.3, has-mir-135b.2, has-mir-135b.1, has-mir-135b, has-mir-1293 and has-mir-1269a were differential miRNA; cg03346179, cg01168201, cg10881514, cg11399097, cg0225127, cg138947963, cg07072722, cg02527199, cg07055504 and cg12887711 were differential methylation sites; chr1.164893737–164946735, chr1.165046515–165656104, chr9.38479129–38619151.1, chr9.33496157–33500957, chr9.91938863–91981877.3, chr9.84521603–84808278, chr9.85166912–85798903.3, chr9.92684729–92818359.1, chr9.111555580–111726070 and chr9.110002756–110379567.3 were the most differential copy number variation sites; SAMD3, THBS2, LOXL3, PRKCQ, FNBP1L, SEC24A, ROCK2, RELN, SECISBP2L and MET were the most differential mutated genes. The MOICS2 subtype presented a favorable prognosis, both in OS and PFI, when compared with the MOICS1 subtype (Figure 1(f), Table S2). The detailed clinical characteristics between subtypes are illustrated in Figure S2(a). In addition, our remodeling results improved the established clinical stratification systems, since MOICS1 and MOICS2 reached a high consistency with those characteristics (Figure S2(b)).

(a) The optimal cluster number of immune subgroups in the TCGA-KIRC cohort; the blue line represents CPI, and the red line represents gap statistics. (b,c) Consensus heatmap based on 10 integrative clustering algorithms and Silhouette plot based on the consensus ensemble results. (d) Comprehensive heatmap of multiomics integrative clustering with a dendrogram for samples. (e) Kaplan‒Meier survival curve of OS (left) and PFI (right) of immune subtypes.

### MOICS1 led a distinctive immune-hot phenotype than MOICS2

**Figure 2.**
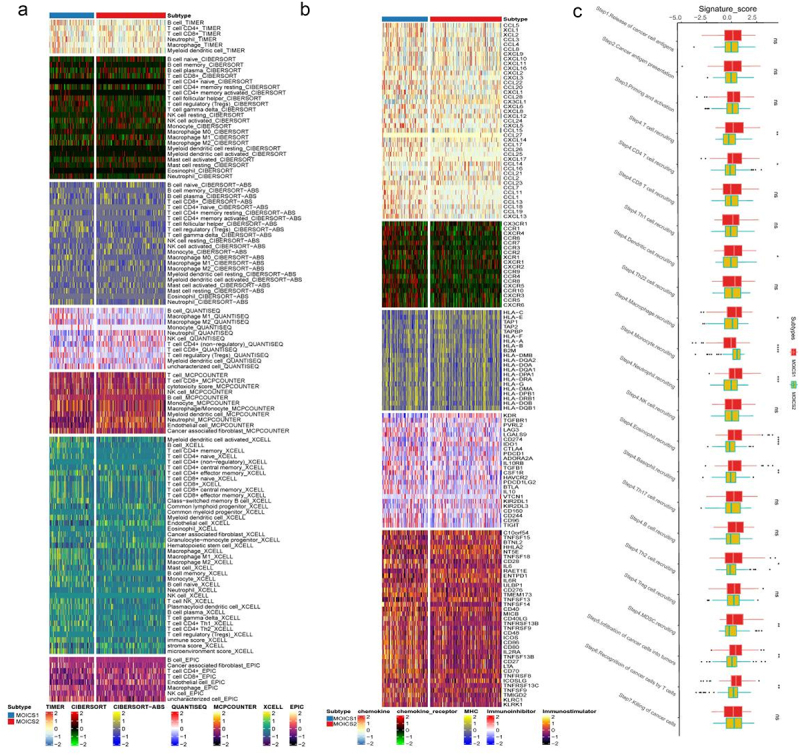
Immune landscapes between the subgroups.

**Figure 3.**
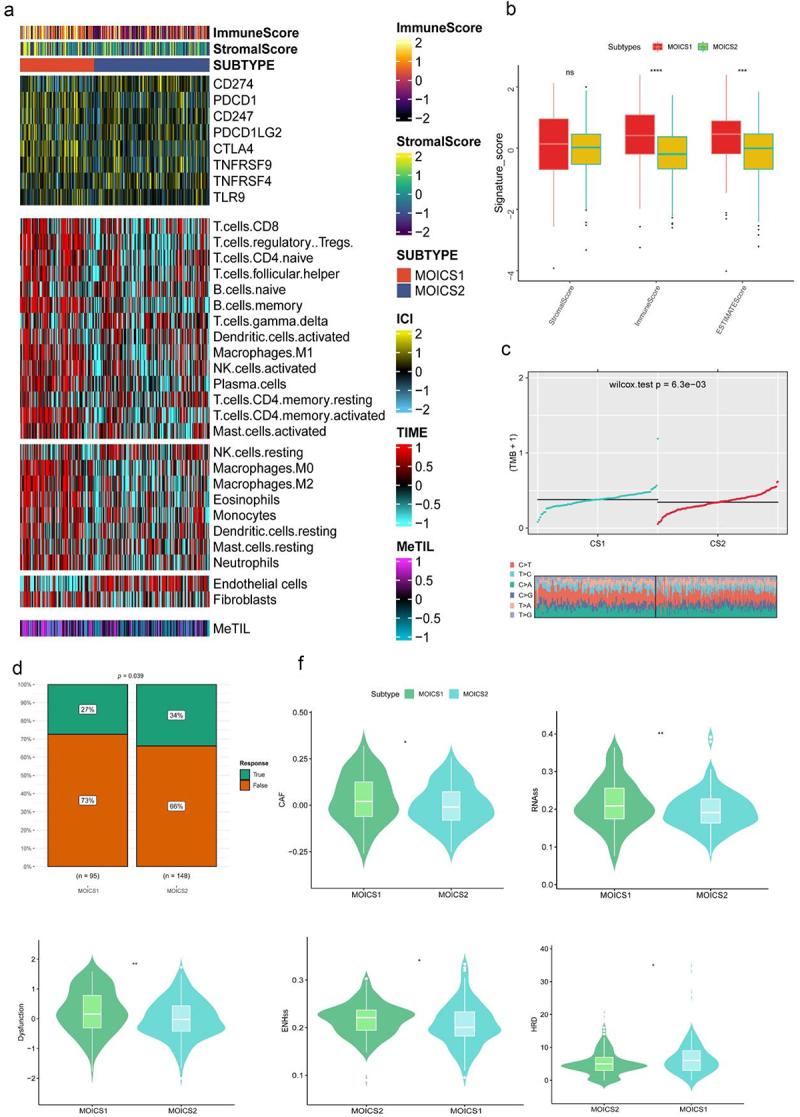
Landscapes of specific immune components and immune function scores.

This stratification was based on an immune-related signature. Herein, we systematically compared the immune components and tumor immunity between subtypes. The results indicated that MOICS1 presented an immune-hot subgroup compared with MOICS2 (Figure 2(a)), since most immune components except of neutrophil-QUANTISEQ, neutrophil-MCPCOUNTER, endothelial cell-MCPCOUNTER, endothelial cell-EPIC and hematopoietic stem cell-XCELL signatures in MOICS1 were higher than those in MOICS2. The expression of immune microenvironment signature-related genes was also significantly different between the two subgroups (Figure 2(b)), except that several chemokines (CLL28, CX3CL1, CCL2, CCL14), MHCs (HLA-C, HLA-R, TAP1, TAP2), immune inhibitors (KDR, CD274, ADORA2A) and immune-stimulators (TNFSF15, NT5E, TNFSF18, ENTPD1, IL6R, RNFSF13 and ICOSLG) were higher in the MOICS2 group. MOICS1 maintained a higher degree of T cells, B cells, macrophages, activated NK cells, mast cells, eosinophils, monocytes, neutrophils, and fibroblasts, while resting NK cells and endothelial cells were more enriched in MOICS2. Through TIP algorithms, we found that most tumor immunity processes in the MOICS1 group were more activated than those in the MOICS2 group, while the monocyte recruitment score was higher in the MOICS2 group (Figure 2(c)). We also found that the expression levels of nine immune check inhibitor signatures, which were used to predict ICIs in clinical practice, were significantly varied between subtypes (Figure S3(a)). The immune infiltration results calculated through the ssG-SEA algorithm were consistent with prior results (Figure S3(b)). The immune score calculated based on deconvolution algorithms, including immune scores, estimate score and DNA methylation of tumor-infiltrating lymphocytes (MeTIL) scores, was also higher in the MOICS1 subgroups (Figure 3(a)). These results indicated that the MOICS1 subgroup might belong to immune-hot renal cancer, while the MOICS2 subgroup might belong to immune-cold renal cancer. The ESTIMATE algorithm further validated this conclusion, since MOICS2 presented higher immune and ESTIMATE scores (Figure 3(b)). In addition, the TMB in the MOICS1 group was higher than that in the MOICS2 group (Figure 3(c)). Regarding the immune response, the MOICS2 subgroups had a higher response rate (Figure 3(d)). The cancer-associated fibroblast enrichment scores, RNA expression-based stemness index (RNAss), tumor immunity dysfunction score and enhancer element stemness score (ENHss) in the MOICS1 group were higher than those in the MOICS2 group, while the homologous recombination deficiency (HRD) score was reversed between subgroups (Figure 3(f)).

(a,b) Heatmap of tumor-related infiltrating immune cells and immune signatures between subgroups. (c) Difference in TIP-related signatures between MOICS1 and MOICS2.

(a) Heatmap showing the immune profile, including immune checkpoint targets, immune enrichment score, stromal enrichment score and DNA methylation of tumor-infiltrating lymphocytes (MeTILs), in the KIRC-TCGA cohort. (b) Stromal, immune and ESTIMATE scores between subtypes. (c–f) Differences in TMB, response to ICI treatment, CAFs, RNAss, dysfunction, ENHss and HRD between MOICS1 and MOICS2.

### Activation of hypoxia signaling in MOICS1 resulted the inferior prognosis

**Figure 4.**
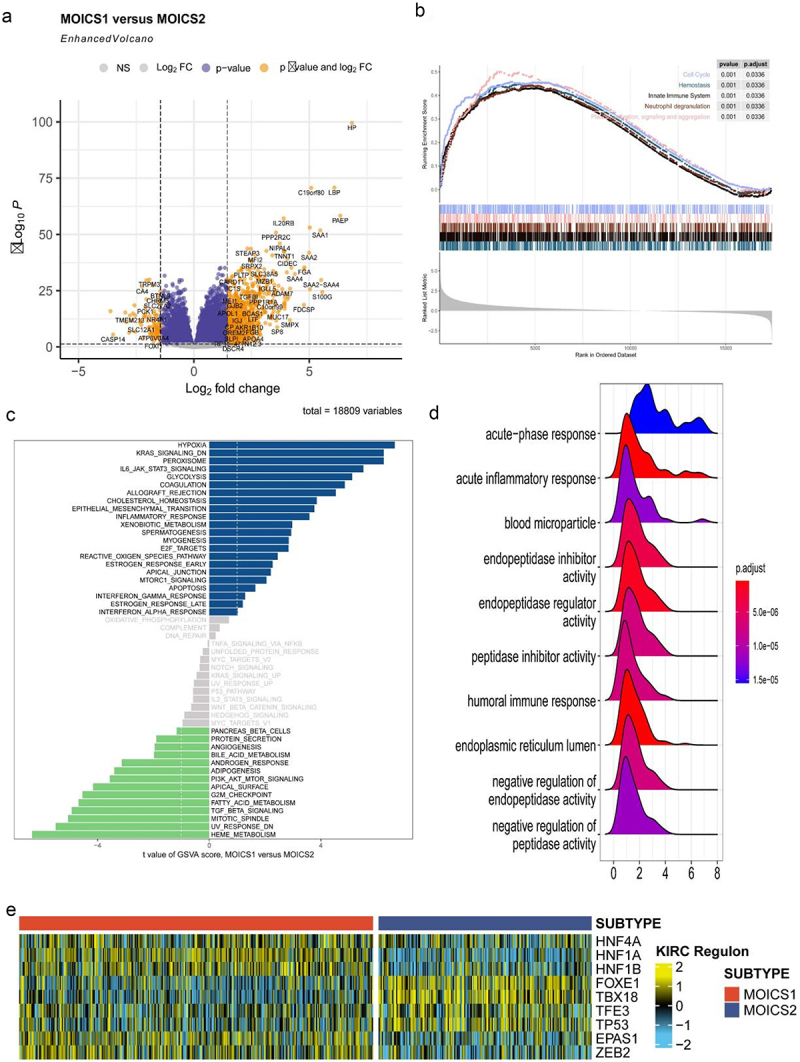
Functional enrichment analysis of ccRCC subtypes.

Based on the clinical and immune differences between subgroups, we next wanted to explore the biological process involved in such differences. In total, 876 DEGs, including 794 upregulated and 82 downregulated genes, were identified between MOICS1 and MOICS2 (Figure 4(a)), and subtype biomarkers are indicated in Table S3. Functional analysis indicated differences in the negative regulation of endopeptidase activity and peptide activity, antimicrobial humor response, cornification and negative regulation of proteolysis in the biological process (BP) module; collagen-containing extracellular matrix, blood microparticles, high-density lipoprotein particles, plasma lipoprotein particles and lipoprotein particles in the cellular compartment (CC) module; and peptidase inhibitor activity, endopeptidase regulator activity, peptidase regulator activity, endopeptidase inhibitor activity and serine-type endopeptidase inhibitor activity in the molecular function (MF) module (Figure S4). Cell cycle, hemostasis, innate immune system, neutrophil degranulation and platelet activation, signaling and aggregation were activated in the MOICS1 subgroups (Figure 4(b)). Through GSVA analysis, we found that hypoxia, KRAS signaling, peroxisome, IL6-JAK-STAT3 signaling, glycolysis and coagulation were activated in the MOICS1 subgroup, while heme metabolism, UV response, mitotic spindle, TGFβ signal and fatty acid metabolism were activated in the MOICS2 subgroup (Figure 4C). KEGG pathway analysis revealed that the acute phase response and inflammatory response were activated in the MOICS1 group (Figure 4(d)). In addition, we also investigated the regulon activity of renal cancer-related transcription factors between subgroups, which indicated that HNF4A, HNF1A, HNF1B, EPAS1 and ZEB2 were activated in the MOICS1 subgroup, while FOXE1, TBX18, TFE3 and TP53 were activated in the MOICS2 subgroup (Figure 4(e)).

(a) Volcano map of DEGs. (b–d) GSEA, GSVA, and KEGG analysis show the hallmarks between subgroups. (e) Heatmap showing profiles of regulon activity for renal cancer-related regulators.

### MOICS1 is characterized as a genomic instability phenotype

**Figure 5.**
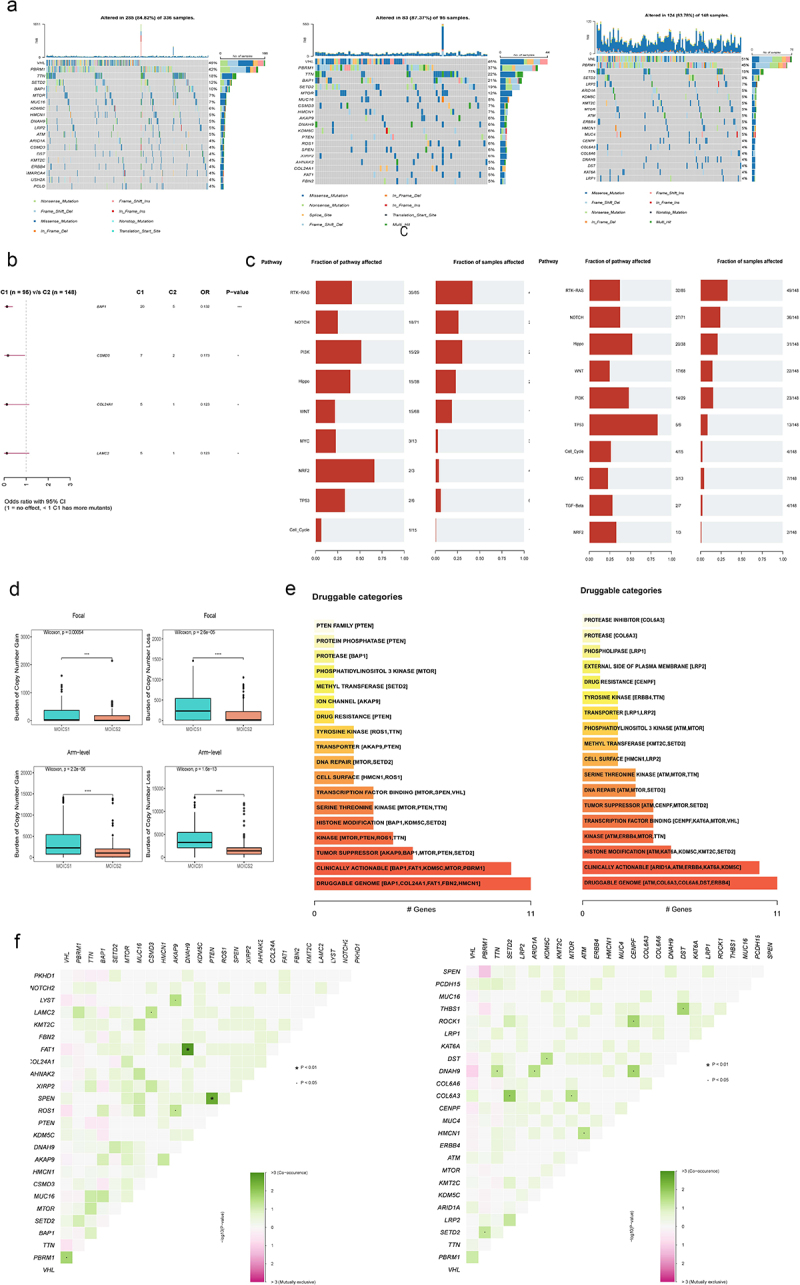
Landscapes of somatic mutations and potential targets between subtypes.

We found that there were different mutation landscapes between MOICS1 and MOICS2. The most common mutated genes are depicted in Figure 5(a). Among them, the mutation rates of VHL, PBRM1, SETD2, TTN and MTOR in the MOICS1 subgroup varied with those in the MOICS2 subgroup (46% vs. 51%; 37% vs. 45%; 19% vs. 9%, 22% vs. 15%, 12% vs. 5%) (Figure 5(a)). BAP1, CSMD3, COL24A1 and LAMC2 were protective mutation genes in the MOICS1 subgroup compared with the MOICS2 subgroup (Figure 5(b)). The carcinogenic pathway between subgroups also varied, among which the frequencies of RTK-RAS, NOTCH, PI3K, Hippo and WNT were different between MOICS1 and MOICS2 (40/96 vs. 49/148, 24/95 vs. 36/148, 29/95 vs. 23/148, 22/95 vs. 31/148, 18/95 vs. 22/148) (Figure 5(c)). Interestingly, the copy number mutation, including focal and arm-level mutations, was higher in the MOICS1 subgroup (Figure 5(d)). Through the function of drug-Interactions in the Maftools package, possible therapeutic targets in MOICS1 included BAP1, COL24A1, FAT1, FBM2 and HMCN1, while MOICS2 mainly contained ATM, COL6A6, COL6A6, DST and ERBB4 (Figure 5(e)). Next, facilitated with the CoMEt algorithm, we found that the specific co-occurring mutations in the MOICS1 subtype included VHL-PRRM1, CSMD3-LAMC2, AKAP9-LYST, AKAP9-FAT1, AKAP9-ROS1, DNAH9-FAT1 and PTEN-SPEN, and there were specific co-mutations, including PBRM1-SETD2, TTN-DNAH9, SETD2-COL6A3, ARID1A-DNAH9, KDM5C-DST, MTOR-COL6A3, ATM-HMCN1, GENPF-ROCK1, CENPF-DNAH9 and DST-THBS1, in MOICS2 (Figure 5(f)).

(a) Waterfall plot showing the top 20 most frequently mutated genes. (b) Forest analysis indicating different mutated genes between the two subtypes. (c) The fraction of pathways or samples of oncogenic signaling pathways in MOICS1 and MOICS2. (d) Potential druggable gene categories from the mutation dataset in MOICS1 and MOICS2. (e) Synthetic lethal mutations in MOICS1 and MOICS2. (f) Waterfall plot showing the mutation patterns of copper-induced cell death-related genes in MOICS1 and MOICS2.

### Frequent CNV and 3q amplification were noticed in MOICS1

**Figure 6.**
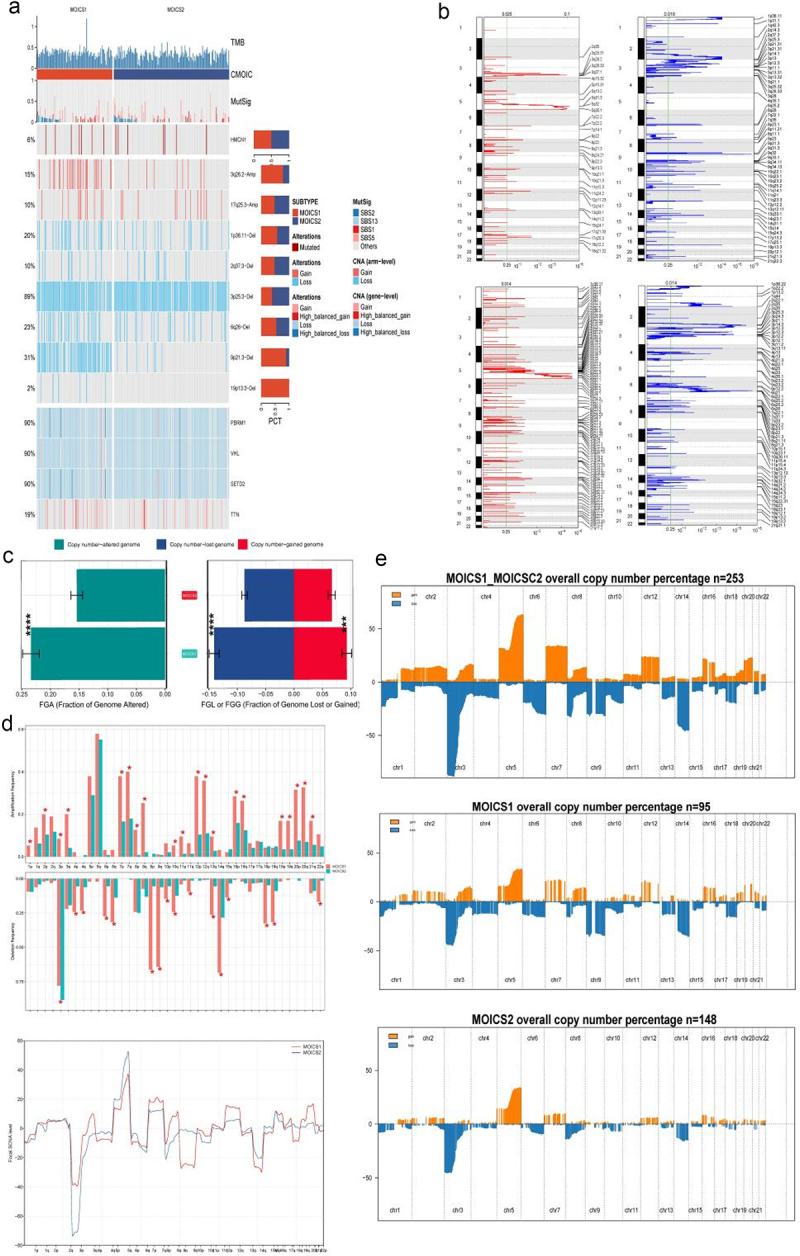
Landscapes of copy number variations between subtypes.

The landscape of mutation differences between MOICS1 and MOICS2 is illustrated in Figure 6(a). CNV differences were compared between MOICS1 and MOICS2. The rate of CNV frequency was higher in MOICS1, both in copy number loss and gain level (Figure 6(a)). The detailed recurrently amplified and deleted regions are shown in Table S4. Through different analyses of CNVs, we found a high frequency mutation of tumor suppressor genes (e.g., VHL and PBRM1) and metabolism regulators (e.g., COL4A3 and COL4A4) between MOICS1 and MOICS2 (Figure 6(b)). The recurrent amplifications of focal CNVs in MOICS1 were located at 3q26.33 (ACTL6A, NDUFB5, USP13 and MRPL47) and 5q32 (PRELID2), and 9p21.3 (C9orf53 and CDKN2A), 9p23 (PTPRD) and 11q21 (CWC15) were deleted. Recurring focal CNVs in MOICS2 included amplifications of 5q35.1 (BNIP1, CANX, CLTB, NKX2–5, DBN1, DOCK2 and DRD1), 16p13.3 (RBFOX1), and 5q33.2 (SGCD) and deletions of 6q25.2 (RGS17 and MTRF1L), 1q44 (ZNF695) and 2q35 (ABCA12). These specific CNVs might contribute to the heterogeneity between subtypes (Figure 6(c)). In addition, there were significant differences in amplification in ch1q, 2p, 3p, 3q, 7p, 7q, 8p, 8q, 10q, 11p, 12p, 12q, 13q, 16p, 16q, 19p, 19q, 20p, 20q and 21q and deletion in 3p, 4p, 4q, 6p, 6q, 10p, 10q, 11q, 13q, 14q, 15q, 18p, 18q and 22q (Figure 6(d,e)). All these results indicated that genomic loss and gain might contribute to the difference in immune heterogeneity between MOICS1 and MOICS2.

(a) Genomic alteration landscape according to immune subtype. (b) Distribution of fraction genome altered (FGA) between subtypes. (c) Comparisons of arm-level and focal-level mutations between subgroups. (d) Bar plot of fraction genome alteration in MOICS1 and MOICS2.

### Axitinib is more suitable for MOICS1 and erlotinib more appropriate for MOICS2

**Figure 7.**
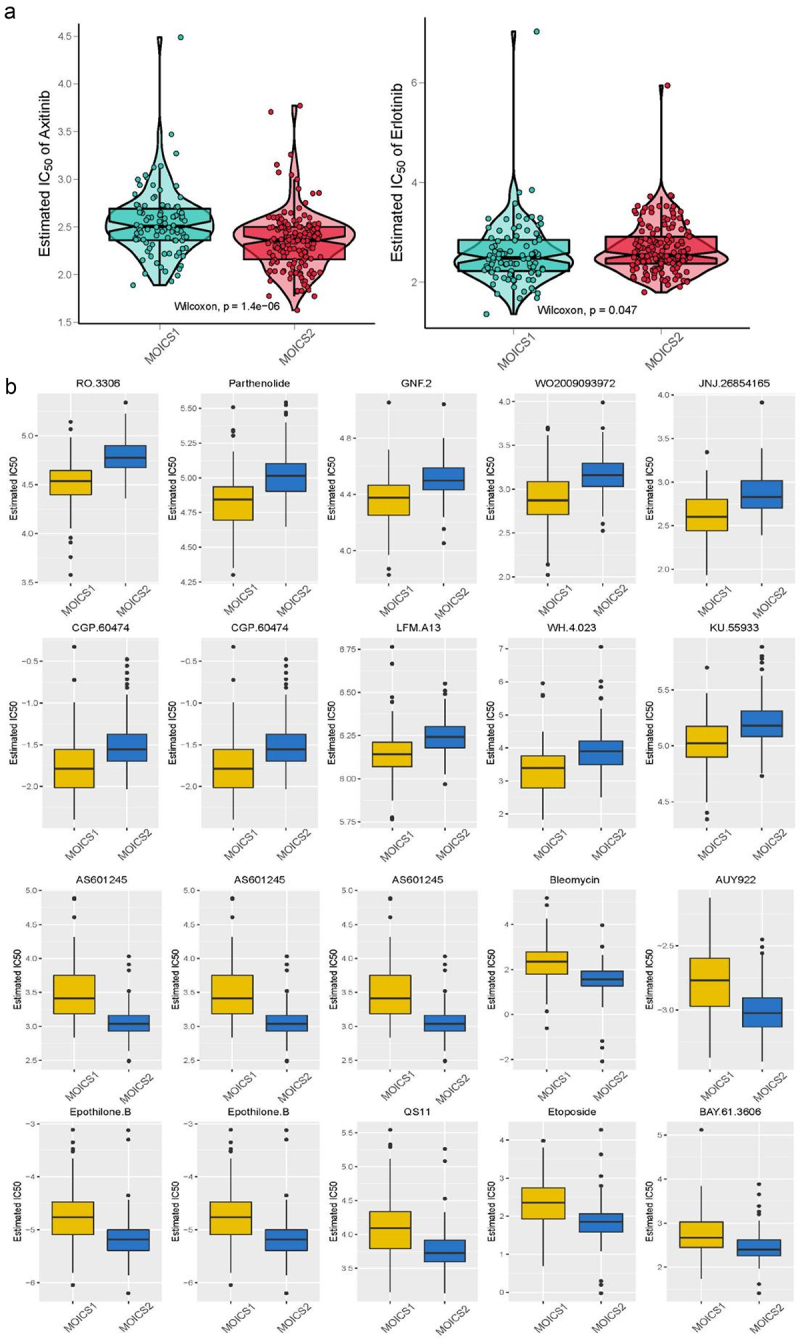
Drug sensitivity analysis of the two subtypes.

In this section, we investigated whether MOICS1 and MOICS2 maintained differences in targeted therapy and found that MOICS2 was sensitive to axitinib, while MOICS1 was sensitive to erlotinib (Figure 7(a)). Since the MOICS1 subtype presented an unfavorable prognosis compared with the MOICS2 subtype, we aimed to investigate the potential targets of MOICS1. The detailed differences in drug sensitivity between MOICS1 and MOICS2 are shown in Table S5. The top potential therapeutic agents for MOICS1 included RO.3306, parthenolide, GNF.2, WO2009093972, JNJ.26854165, CGP.60474, temsirolimus, and LFM. A13, WH.4.023, and KU.55933; and the MOICS2 subgroup was sensitive to AS601245, GSK.650394, vinorelbine, bleomycin, AUY922, and epothilone. B, QS11, FH535, etoposide, and BAY61.3606 (Figure 7(b)).

(a) Differences in the estimated IC50 of axitinib and erlotinib between MOICS1 and MOICS2. (b) Different sensitivity, IC50, to chemotherapy between subtypes.

### Remodeling results reached robustness in out-cohort and immune cohort

**Figure 8.**
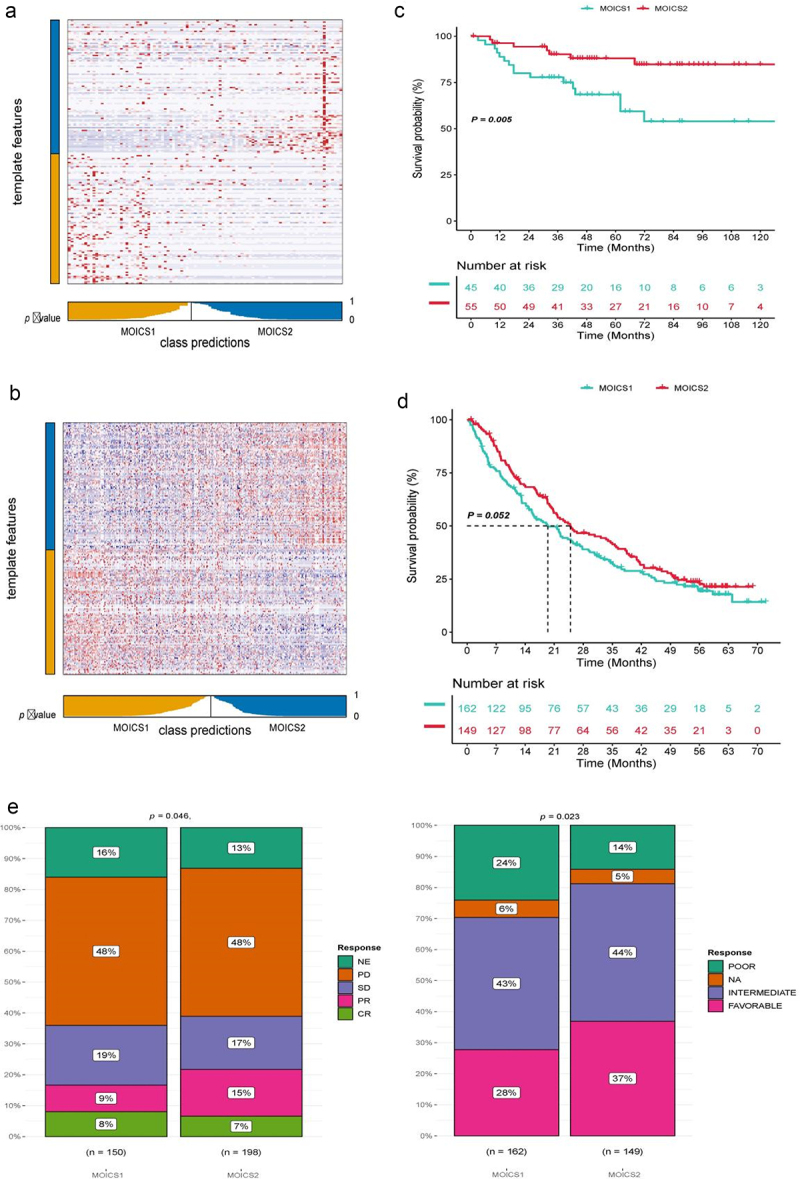
Verification of the classification model in out-house datasets.

We constructed a well-performing immune-related classifier in the previous sections. We then aimed to evaluate the reproductivity of such a classifier in outhouse cohorts, which included the JAPAN-KIRC and immune therapy cohorts. To better apply our classier in clinical practice, we extracted subtype-specific markers with the use of function runMarker from MOVICS package. Next, the nearest template prediction (NTP) algorithm was utilized to classify out cohort datasets and divide patients into MOICS1 and MOICS2 according to subtype biomarkers mentioned before. We found that patients in each cohort could be reclassified into the MOICS1 and MOICS2 groups (Figure 8(a,b)). The prognosis of subtypes in JAPAN-KIRC was similar to that in the discovery cohort in TCGA-KIRC (*p* = .005) (Figure 8(c)) and in Braun’s cohort (*p* = .126) (Figure 8(d)). In addition, the clinical characteristics of subtypes in Braun’s cohort were significantly different, while patients classified into the MOICS2 subgroup had a higher response rate than those classified into the MOICS1 subgroup (Figure 8(e)). In patients with advanced stage disease, our remodeling system also reached a good sensitivity and specificity to evaluate patients’ clinical outcome, since the MOICS1 subtype in advanced ccRCC or urothelial carcinoma patients received or did not receive immune and target therapy, and such consistency could be detected at the mRNA and protein levels in ccRCC (Figure S5(a–c)).

(a) Heatmap of NTP in the JAPAN-KIRC cohort via subtype-specific biomarkers from MOICS1 and MOICS2. (b) Survival analysis of the two predicted subtypes of ccRCC in the JAPAN-KIRC cohort. (c) Heatmap of NTP in the ICI cohort via subtype-specific biomarkers. (d) Survival analysis of the two predicted subtypes of ccRCC in the immunotherapy cohort. (e) Immune therapy response difference in the immunotherapy cohort.

### A satisfactory and reproductive risk model constructed by subtypes’ biomarkers

**Figure 9.**
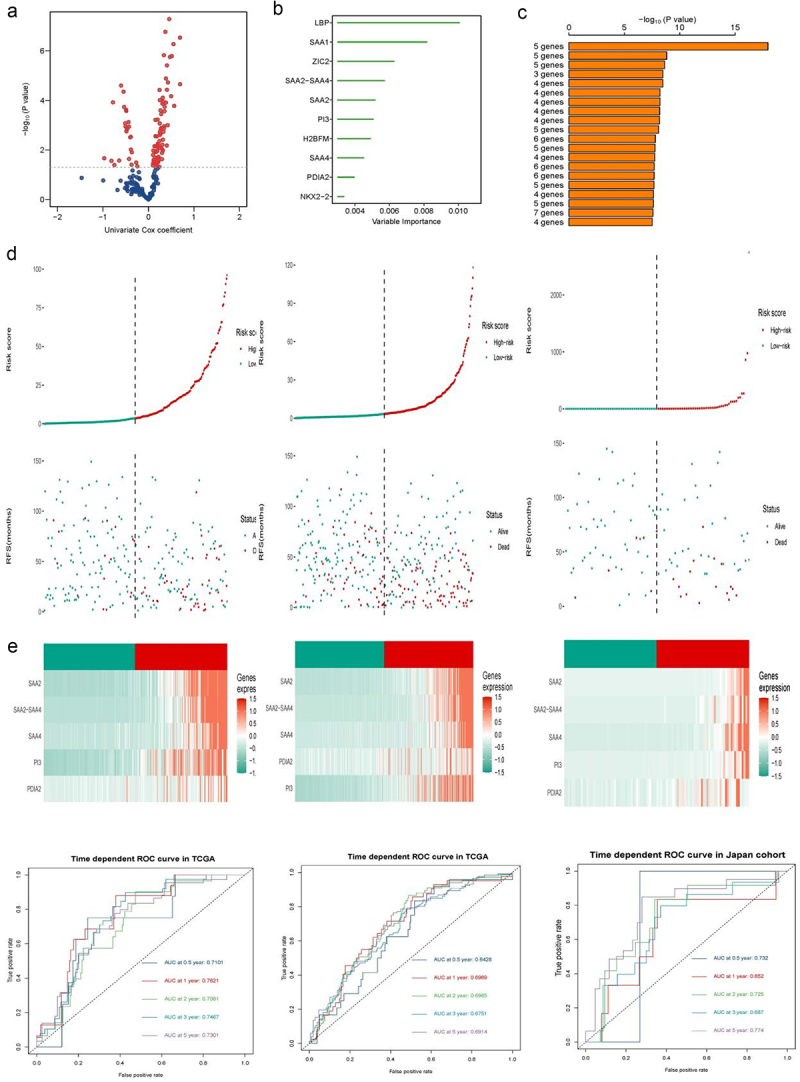
Construction and validation of a subtype biomarker based on the risk model.

n this part, we aimed to construct and verify a model based on the subtype biomarkers mentioned above. We utilized univariable Cox regression algorithms to filter those signatures associated with patient OS first (Figure 9(a)). The prognostic weight of each biomarker is ranked in Figure 9(b). We introduced RSFVH algorithm to identify the most concise and robust prognostic model (Figure 9(c)). Finally, a five-gene (SAA2-SAA4, SAA2, PI3, SAA4, PDIA2) composed prognostic model was constructed, and the detailed risk calculated formula was as follows: Risk score = 4.776701* SAA2-SAA4 + 4.793939*SAA2 + 4.123623* PI3 + 3.260562*SAA4 + 3.433174*PDIA2. We calculated each single patient risk score in the training and other two test cohorts and divided patients from each group into high- and low-risk subgroups by comparison with the median risk score (Figure 9(d), Figure S). The prognostic value of this risk model was also evaluated via a time-dependent ROC curve, which reached a satisfactory result in multi ccRCC cohorts (Figures 9(e) and S6(a–c)).

(a) Volcano plot showing the subtype-based biomarkers by univariable Cox regression analysis. (b) Random survival forest analysis screening 10 genes. (c) Based on various combination analyses, the top signatures are ordered by the *p* value. (d) Risk score analysis in the TCGA-KIRC training, TCGA-KIRC test and Japan-KIRC cohorts. (e) Time-dependent ROC curves for the risk signatures in the three cohorts.

### Dysregulated expression level of SAA4 promotes the progression of ccRCC

**Figure 10.**
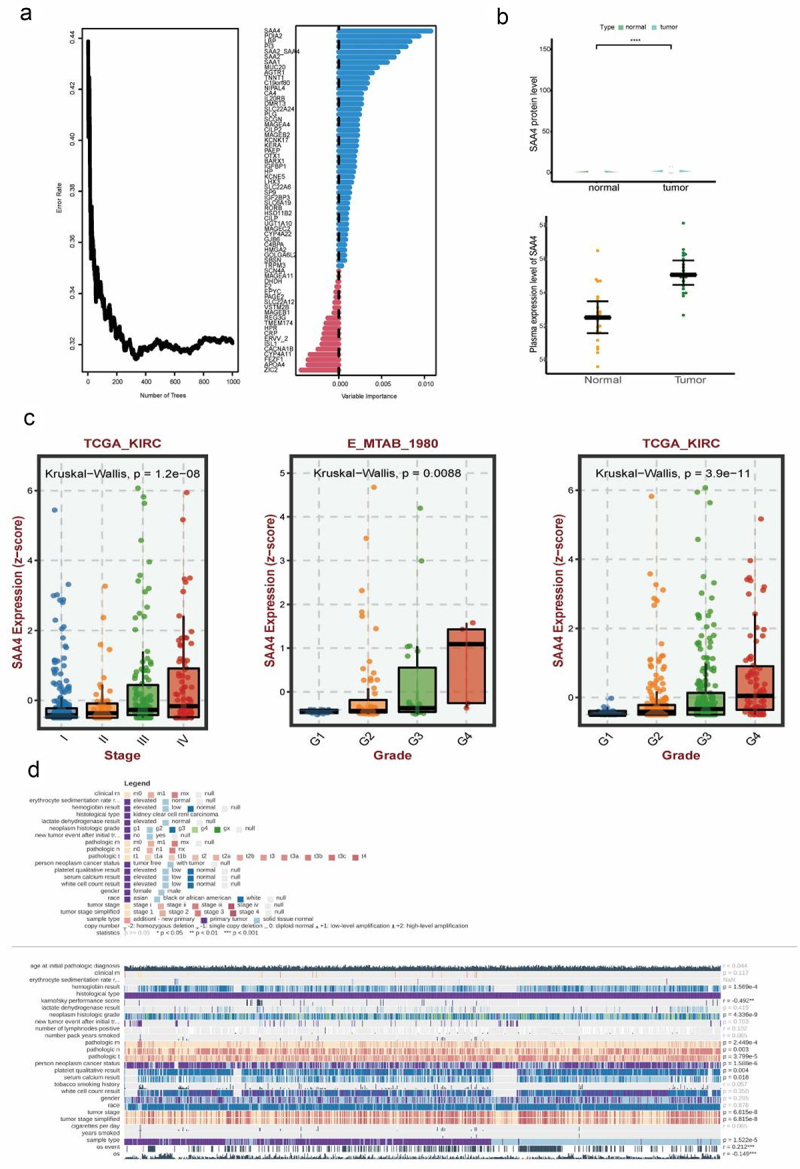
The hub role of SAA4 in ccRCC.

Previous results revealed that subtype MOICS1 had an inferior clinical outcome and was validated in multiple ccRCC cohorts. Thus, we aimed to identify the leading signature in such a subtype based on machine learning. Among the top biomarkers from two subgroups, random forest-based regression analysis showed that SAA4 displayed the highest proportion of significance in ccRCC (Figure 10(a)). Aided by proteogenomic datasets, we found that SSA4 was highly expressed in tumor tissues and late-stage patients and validated the aberrant SAA4 expression level in patient serum by ELISA (Figure 10(b)), which was also detected at the transcriptome level (Figure 10(c)). Among ccRCC cohorts with clinical outcome information, SSA4 functioned as a risk factor; based on median expression, we found that the high SAA4 expression subgroup displayed shorter survival than the low subgroup (Figure 10(d)). Additionally, we have identified that high expression levels of SAA4 across multiple datasets are a prognostic risk factor for patients with ccRCC (Figure S7(a,b)). All these findings suggest that SAA4 might be treated as a novel prognostic and noninvasive biomarker in ccRCC.

(a) Random forest indicating the prognostic impact of biomarkers of MOICS1. (b) Differential expression of SAA4 in the ccRCC patient cohort at the protein level. (c) SAA4 expression level varies in normal, tumor tissue and different stage and grade ccRCC tumor tissues. (d) Clinical outcome impact of SAA4 in ccRCC.

In addition, guilty of association analysis reminded us that SAA4 might involve in acute phase response and inflammation response in GO and activate IL6-JAK-STAT3 signal in ccRCC (Figure S8(a,b)). Interestingly, when adding SAA4 protein (HY-P71277, MCE) in to medium of RCC cell lines including 7860 and ACHN, we noticed that the malignant ability of both cell lines was enhanced when comparing to control group (Figure S6(c-e)). In pan-cancer level, we noticed that SAA4 was dysregulated in several cancer types including HNSC, KIRP, COAD, READ, LIHC, BRCA, KICH, THCA, LUAD, CHOL, ESCA and STAD (Figure S9(a)). High expression level of SAA4 predicted a worse prognosis in OS among cancers including CHOL, KIRC, KIRP, LGG, OV and PAAD (Figure S9(b)). To confirm whether SAA4 display a consistent biological role among cancer, we performed GSEA analysis across cancers. As Figure S9(c) showed that SAA4 might participate in TNFα-NFκB, KRAS, interferon, inflammatory, IL6-JAK-STAT3, IL2-STAT5 signals in most cancer types.

## Discussion

RCC represents 3% of adult malignancies worldwide, positioning it among the most prevalent cancers.^36^ ccRCC, the most common clinical subtype, is notably associated with diverse clinical prognoses and demonstrates metastasis at diagnosis in up to 30% of cases. Traditionally, the primary treatment for localized ccRCC has been radical or partial nephrectomy.^37^ Despite significant advancements in surgical techniques over recent decades, about 30–40% of patients still experience metastasis or relapse after surgery, and the survival rate for localized stage III patients drops to 53%.^38^

ccRCC is characterized by a highly inflamed tumor microenvironment, primarily due to the infiltration of immune cells and the low cellular purity of the tumors.^39^ This immunological landscape has positioned ccRCC as one of the first solid tumor cohorts to benefit from immune therapy.^40^ The encouraging outcomes from several clinical trials have propelled immune therapy to the forefront of ccRCC management.^41^ However, only a fraction of patients derives significant benefits from such treatments,^42–44^ and the absence of effective biomarkers to identify immunotherapy-responsive patients, coupled with severe adverse events, continues to restrict the broader application of ICIs. Consequently, the identification of mechanisms and therapeutic targets that drive ccRCC progression is of paramount importance, not only to enhance therapeutic efficacy but also to tailor treatments to individual patient profiles, potentially transforming outcomes in this challenging cancer subtype.

The molecular characteristics of ccRCC based on single omics or specific gene lists have been extensively studied.^15,20^ A single omics expression matrix might lose the essential omics characteristics, while aggregating different omics data via the consensus ensemble principle could provide new insight into decoding the heterogeneity of ccRCC. Fortunately, the development of high-throughput sequencing and bioinformatics promotes the elucidation of comprehensive molecular alteration landscape in ccRCC. Many novel risk stratification schemas were established based on different altered molecular and forms. Motzer and colleagues performed an integrative multi-omics analysis on 823 RCC specimens obtained from a randomized clinical trial.^45^ Employing transcriptional profiles and gene alteration data, they constructed a robust molecular classification framework. This stratification, underpinned by varied responses to VEGF blockade used alone or in combination with anti-PD-L1, provides crucial insights for personalizing treatment strategies and guiding future therapeutic advancement in RCC. Through an exhaustive proteogenomic characterization in 103 untreated patient samples of clear cell renal cell carcinoma, Clark et al. illuminated tumor-specific alterations at the proteomic level, previously undisclosed by transcriptomic profiling.^46^ It further advances a refined subtyping schema derived from a thorough omics analysis integration. In an encompassing study by Chen et al., 894 renal cell carcinomas underwent rigorous analysis involving DNA mutation and copy data, DNA methylation, and gene expression. As a result, the cancers were stratified into nine principal subtypes.^47^ Each subtype was distinctly characterized by altered pathways and associations with patient survival. Even previous works have provided some understanding of the heterogeneity of RCC at multi omics level, while the integration approach or the signatures enrolled for further were broad. Heterogeneity of ccRCC at immune spectrum remains largely unknown.

Recent studies have underscored the pivotal role of the TIME in predicting prognoses and shaping responses to immunotherapy in ccRCC. For instance, Rebuzzi et al. demonstrated a strong correlation between the presence of specific immune cell types – particularly CD8^+^ T cells, CD4^+^ T cells, and PD-L1 expression – and patient responses to nivolumab treatment.^48^ This correlation aligns with findings by Brown et al., who identified distinct expression patterns of immune markers differentiating responders from non-responders to ICIs.^49^ These patterns are potentially predictive of treatment outcomes, suggesting that a nuanced understanding of tumor-infiltrating lymphocytes (TILs) could substantially refine the personalization of immunotherapy in RCC. Further emphasizing the complexity of the immune landscape in ccRCC, the comprehensive analyses by de Vries-Brilland et al. highlighted how the density and type of immune infiltrates might dictate therapeutic strategies.^50^ This perspective advocates for biomarker-driven trials that tailor treatment plans based on specific characteristics of the TIME, potentially enhancing patient outcomes. Despite these advancements, the variability in TME composition among patients poses a significant challenge in uniformly predicting treatment responses. Future research should aim to integrate multi-omic data to develop more robust biomarkers capable of accurately predicting and monitoring responses to immunotherapy, thus advancing the field of precision medicine in ccRCC.

In the present study, we identified two distinctive ccRCC immune subgroups by integrating 6 immune-related multiomics datasets and identified two robust integrative consensus subtypes (MOICS1 and MOICS2). Prognostic features, immune infiltration landscapes, biological functions, mutation statuses, and drug responsiveness were compared between the two subgroups. In addition, a robust risk prediction model, which could accurately divide patients into high- or low-risk subgroups, was established and verified.

Our work reflects innovation in several aspects. First, the unsatisfactory response efficiency among ccRCC patients might be ascribed to derangement at the epigenomic level since the genomic instability of ccRCC is below the average level among solid tumors. Therefore, it is urgent to investigate the immune escape mechanisms at the multiomics level. After filtering the immune signature related to the TIME, a robust consensus ensemble was generated, which maintained the most significant signatures of each omics profile. Then, we compared the differences in transcriptomic profile (mRNA, lncRNA and miRNA), DNA methylation, CNAs, and somatic mutation spectrum between subgroups, which revealed distinctive molecular patterns and justification of cluster analysis. Prognostic analysis of these two groups indicated the correlation between distinct subgroups and clinical outcomes.

Next, since the extent of immune infiltration influences the tumor immune microenvironment, we systematically deciphered the immune escape mechanisms between MOICS1 and MOICS2. By analyzing the immune components and tumor immunity between the two subgroups, we proposed that the MOICS1 subgroup was associated with an immune-hot phenotype, which was characterized by more infiltrating cells, high expression of immune-related genes, a higher degree of infiltrating immune cells, a high immune score, and high enrichment of the immune-related signature. In contrast, the MOICS2 subgroup was regarded as having an immune-cold phenotype. Tumor‑infiltrating immune cells can be used to evaluate the immune status. As adaptive immune cells, Tregs can suppress the anti-tumor response and promote immune tolerance.^51,52^ Meanwhile, studies have demonstrated that CAFs can remodel the tumor immune microenvironment by secreting cytokines and chemokines, recruiting immune cells, and suppressing cytotoxic lymphocytes.^53^ In our study, we found that Tregs and CAFs were enriched in MOICS1. Therefore, it was speculated that the enrichment of immunosuppressive cells, including Tregs and CAFs, might facilitate immune evasion in MOICS1.

Tumor immunogenicity is mainly determined by antigen presentation efficiency and influences the response to ICIs.^54^ We attempted to evaluate the efficiency of antigen presentation through the expression levels of human leukocyte antigens (HLAs). As a nonclassical immunoregulatory molecule, HLA-E was significantly upregulated in the MOICS2 group. In the tumor microenvironment, HLA-E exhibits its suppressive immunomodulatory properties through binding receptors CD94/NKG2A and NK cells.^55,56^ A previous study established that the overexpression of HLA-E was frequent and common in RCC and led to impaired immunogenicity.^56^ In addition, immune-inhibitor molecules (such as KDR, CD274, and ADORA2A) were markedly upregulated in MOICS2. This result demonstrated that the lack of immuno-infiltrating cells and dysregulation of intrinsic immune regulatory molecules might lead to the immunosuppressive phenotype in MOICS2, which would ultimately facilitate immune escape in ccRCC.

In addition, functional analyses revealed that the innate immune system, inflammatory response, and interferon γ and α response were consistent with the above results. Meanwhile, we observed that multiple metabolic signatures were enriched in MOICS1, including hypoxia, glycolysis, cholesterol homeostasis, and xenobiotic metabolism. Notably, recent research robustly demonstrates that hypoxia profoundly influences the TIME, precipitating immune dysfunction and attenuating the efficacy of immunotherapies, particularly in ccRCC.^57^ For instance, Liu et al. have shown that HIF1A significantly contributes to T-cell exhaustion in gliomas by upregulating immune checkpoint molecules such as PD-L1 alongside exhaustion-related genes, culminating in a predominance of exhausted T cells.^58^ This observation is critical for understanding hypoxia and cancers, as analogous mechanisms potentially undermine immunotherapy by fostering an immunosuppressive TIME. In a related vein, Vignali et al. investigated how hypoxia-induced CD39 expression on exhausted T cells exacerbates immune suppression by promoting adenosine production, which impedes T-cell proliferation and cytotoxic functions.^59^ This interaction highlights the intricate relationship between hypoxia and metabolic shifts within the TIME, profoundly impacting the outcomes of immunotherapies. Further, Sattiraju et al. detailed how hypoxia configures the TIME, drawing TAMs into hypoxic niches within gliomas, where they adopt a pro-tumorigenic role that fosters tumor progression and survival, a scenario mirrored in RCC where hypoxia similarly recruits immunosuppressive cells, thereby obstructing effective immune surveillance and treatment.^60^ Collectively, these studies elucidate how hypoxia establishes formidable barriers to successful cancer immunotherapy. Through promoting T-cell exhaustion, amplifying immunosuppressive cell functions, and modifying metabolic pathways, hypoxia ensures that the immune system cannot mount a vigorous anti-tumor response, thereby challenging current therapeutic strategies. Intriguingly, this study discovered that the hypoxia pathway is significantly activated in patients with the MOICS1 subtype, alongside marked activation of hypoxia-regulated transcription factors such as HIF1A and HIF1B. Although the MOICS1 subtype exhibits higher immune scores and greater infiltration of most immune cells compared to the MOICS2 subtype (Figure 3(b)), the immune dysfunction score is significantly higher in MOICS1 than in MOICS2 (Figure 3(f)). Additionally, the response rate to ICIs therapy is lower in MOICS1 compared to MOICS2 (Figure 3(d)). These findings suggest that modifying the hypoxic state may benefit the remodeling of anti-tumor immune cell functionality.

Besides, ccRCC was regarded as a metabolic disease, which is characterized by glycogen and lipid accumulation.^61^ Altered glucose, lipid, and amino acid metabolism are highly prevalent in ccRCC, which influences the biological behavior of cancer cells.^62^ Therefore, metabolism-related pathways may be targets in renal cancer. Consistently, we found that endothelial PAS domain protein 1 (EPAS1) was obviously activated in MOICS1. EPAS1 encodes a transcription factor called hypoxia-inducible factor 2-alpha (HIF-2α), which is the driver promoting the development and progression of ccRCC.^63^ Our work revealed potential metabolic targets in the treatment of different immune-related subgroups.

The distinct mutation profiles might lead to oncological signaling pathway activation. By comparing the fraction of samples with genes altered in each pathway, we found that RTK-RAS was the most affected pathway. In recent research, the RTK-RAS pathway was demonstrated to regulate immunotherapeutic sensitivity in glioma patients.^64^ Based on the mutation spectrum, we found that BAP1, COL24A1, FAT1, FBN2, and HMCN1 were potential treatment targets in MOICS1, while ATM, COL6A6, COL6A6, DST and ERBB4 were potential therapeutic targets in MOICS2. These subgroup-specific molecular landscapes may be helpful for optimizing treatment strategies.

Finally, we proposed and verified a robust risk prediction model, which was established depending on subtype-related biomarkers. Our model was composed of five genes, including SAA2, SAA2-SAA4, SAA4, PI3, and PDIA2. It is worth noting that SAA2 and SAA4 played significant roles in the prognosis of ccRCC patients. SAA2 and SAA4 belong to the serum amyloid A (SAA) protein family, which is produced in the liver and elevated in an acute-phase response.^65,66^ SAA can be secreted into blood circulation and bind to high-density lipoprotein particles. The metabolism and transport of cholesterol are influenced by SAA.^67,68^ Ignacio et al. found that SAA could facilitate the formation of an inflammatory TME in triple-negative breast cancer.^69^ In addition, SAA was confirmed to have impacts on metastasis and immune biology in cancer.^70^ Patients with higher levels of SAA2, SAA4, and SAA2-SAA4 displayed worse survival in our model, indicating that these SAA genes could serve as biomarkers of prognosis for patients with ccRCC. Meanwhile, our risk model displayed robust and trustworthy ability in two ccRCC cohorts.

The study also contains some limitations. First, the multiomics datasets utilized in our study were retrieved from external public resources. Second, the risk biomarkers and the role of SAA4 in the ccRCC immune microenvironment should be validated by biochemical experiments. Furthermore, the risk prediction model needs to be validated in our own ccRCC cohort with a larger sample size in future studies. In summary, we identified two immune subtypes with distinctive landscapes using multiple immune-related omics data that precisely stratified clinical outcomes, immune microenvironment characteristics, response differences to first-line therapies and divergence among multiple omics levels. A robust classifier was stable and promising for assessing the outcomes of ccRCC patients, thus providing deep insights for the precise management of ccRCC patients.

## Conclusions

Since the perturbation of the immune related signatures in ccRCC at mulitomics level remain largely elusive. To address such problem, this study utilized partial correlation index to identify the immune signatures at five omics level and applied consensus cluster framework to remodel ccRCC patients. We revealed the paradoxical correlation between immune infiltration and endothelial score, which suggested ccRCC patient with an immune relative cold while angiogenesis hot microenvironment might benefit from immune therapy. The robustness and reproductivity of our remodeling systems were further tested in more 1000 ccRCC cases from independent cohorts, all those finding laid a foundation for future subtype-based targeted interventions.

## Supplementary Material

Supplemental Material

TableS5.csv

TableS3.csv

TableS4.csv

TableS2.csv

TableS1.csv

## Data Availability

Access details of the public datasets used in this work can be found in the methods and materials section of the study.

## Abbreviations

ccRCC: clear cell renal cell carcinoma
MOICS: multiomics immune-based cancer subtype
RCC: Renal cancer cell
TIME: tumor immunological microenvironment
ICIs: immune checkpoint inhibitors
TMB: tumor mutation burden
MSI: microsatellite instability
CNV: copy number variation
CPI: Clustering Prediction Index
DEG: differentially expressed gene
ssGSVA: single-sample gene set enrichment analysis
GO: Gene Ontology
KEGG: Kyoto Encyclopedia of Genes and Genomes
GSEA: Gene Set Enrichment Analysis
GSVA: Gene Set Variation Analysis
TIDE: Tumor Immune Dysfunction and Exclusion
CNA: copy number alterations
GDSC: Genomics of Cancer Drug Sensitivity
IC50: half-maximal inhibitory concentration
RSFVH: Random Survival Forest Variable Hunting
HR: hazard ratio
MeTIL: methylation of tumor-infiltrating lymphocytes
RNAss: RNA expression-based stemness index
ENHSS: enhancer element stemness score
HRD: homologous recombination deficiency
BP: biological process
CC: cellular compartment
MF: molecular function
NTP: nearest template prediction
OS: Overall survival
PFS: Progression-free survival
TBR: TGFβ response
HLA: human leukocyte antigens
EPAS1: endothelial PAS domain protein 1
HIF-2α: hypoxia-inducible factor 2-alpha.
