# Supplementary material for: MOICS, a novel classier deciphering immune heterogeneity and aid precise management of clear cell renal cell carcinoma at multiomics level
Source: Cancer Biol Ther. 2024 Apr 24;25(1):2345977. doi: 10.1080/15384047.2024.2345977 (PMC11057626; doi:10.1080/15384047.2024.2345977)
Supplement: Supplemental Material [file KCBT_A_2345977_SM0288.docx]

**Supplementary Figures**

**
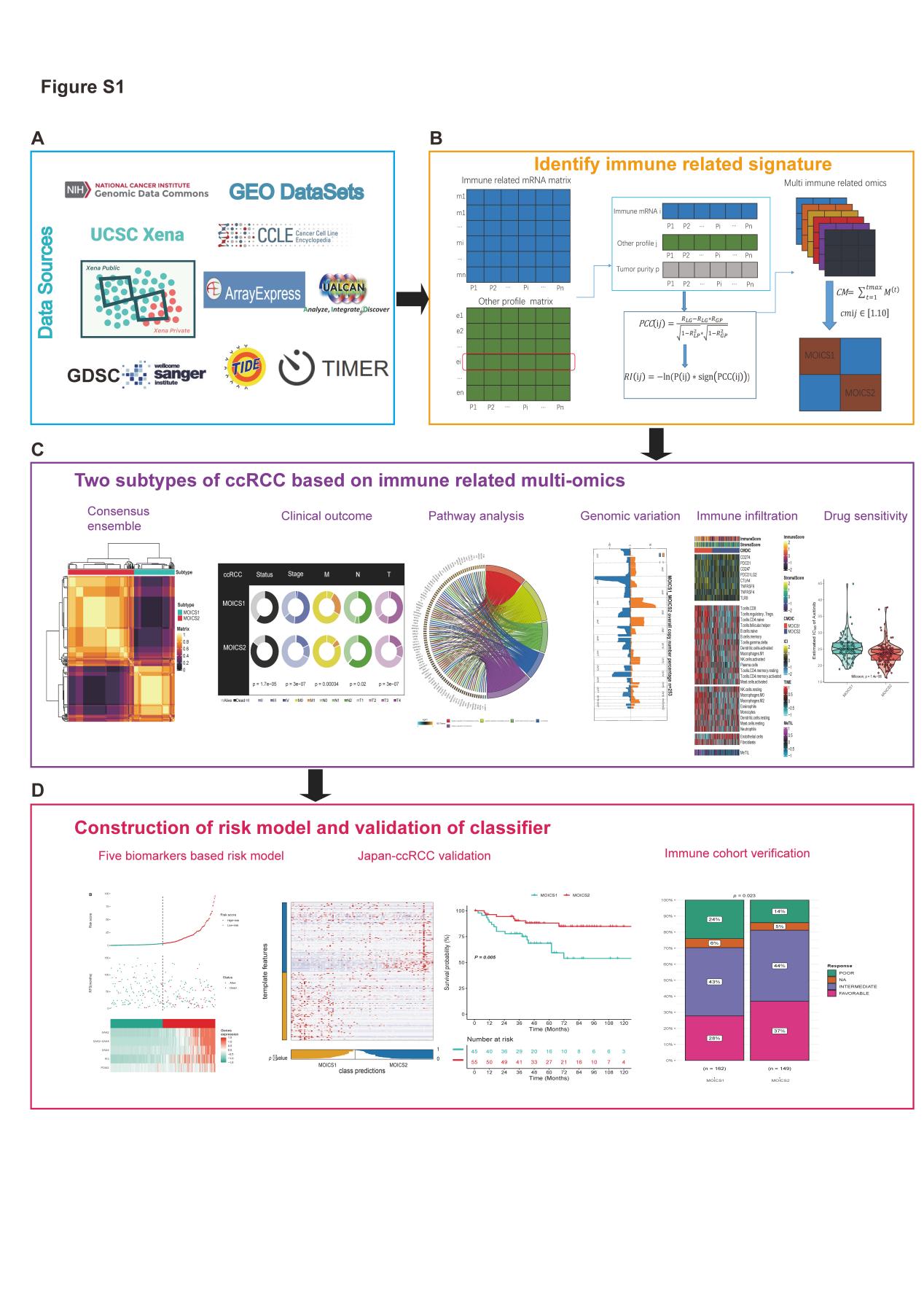
**

**Figure S1** The pipeline of this study.


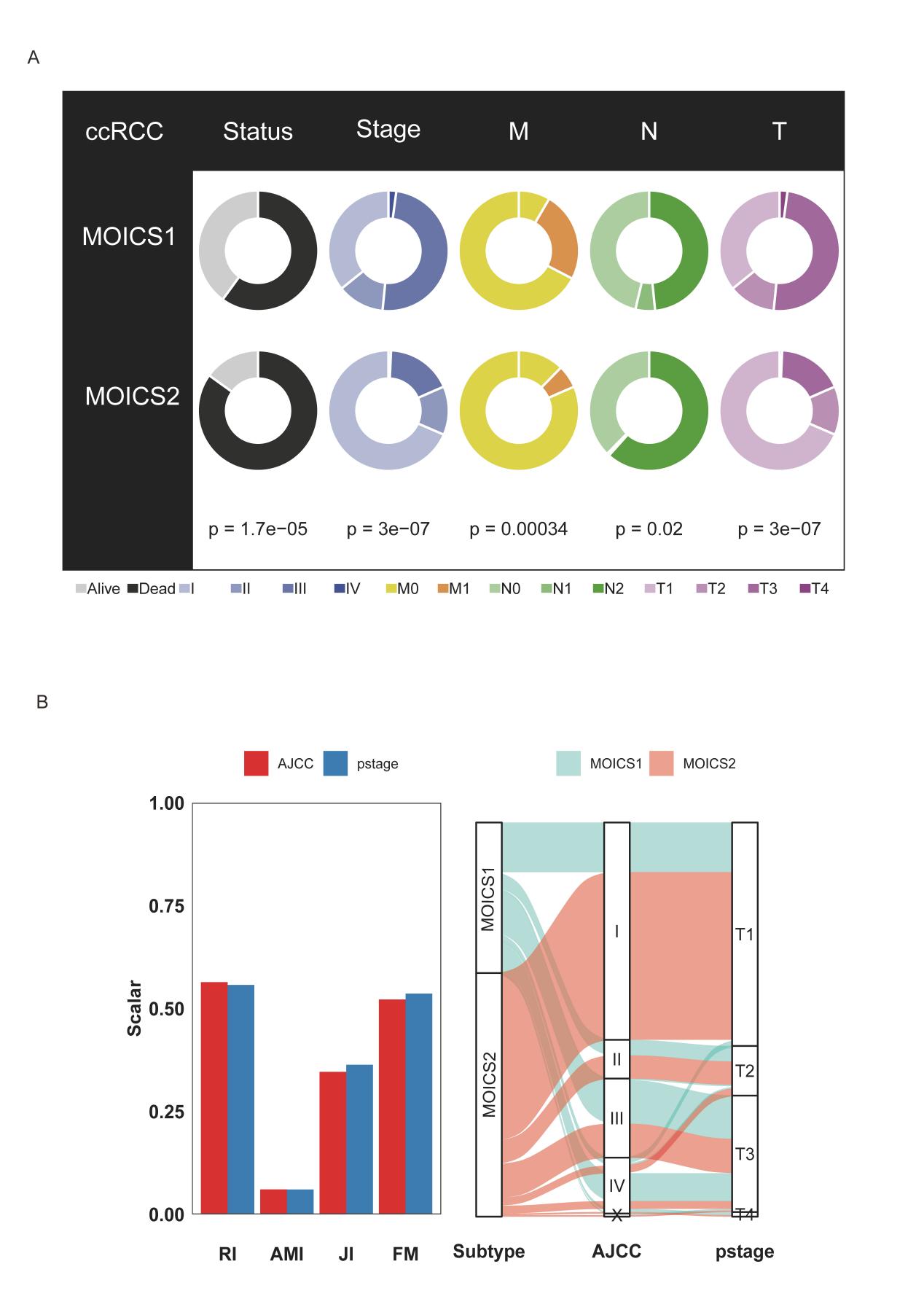


**Figure S2** (A) Different distribution of clinical features of ccRCC patients between subtypes. (B) Agreement of the immune remodelling classifier with the clinical stratification system.


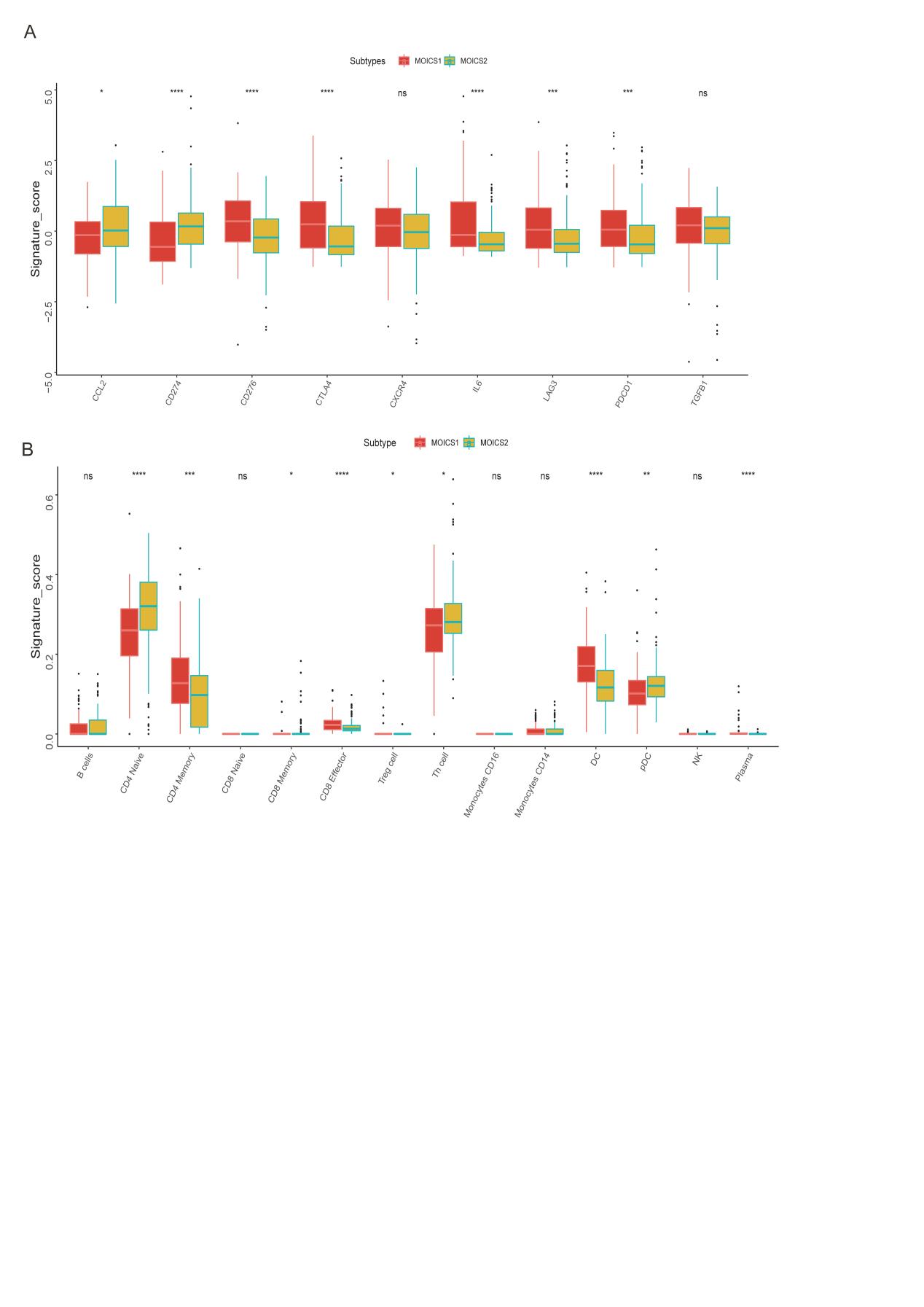


**Figure S3** (A-B) Differential expression of immune checkpoint inhibitors and normalized enrichment scores of immune cells between subgroups.


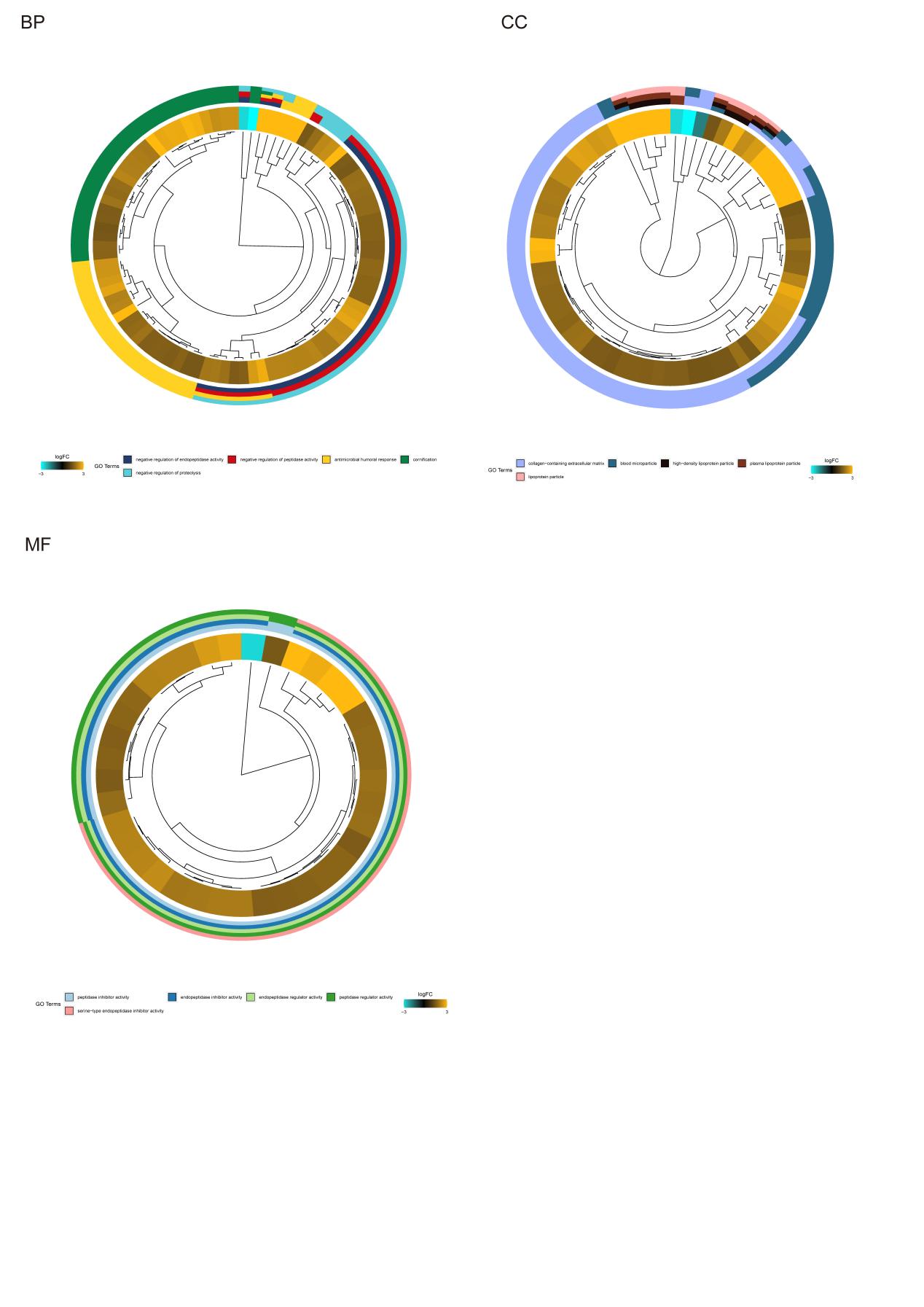


**Figure S4** GO enrichment analysis between subtypes, including BP, CC, and MF.


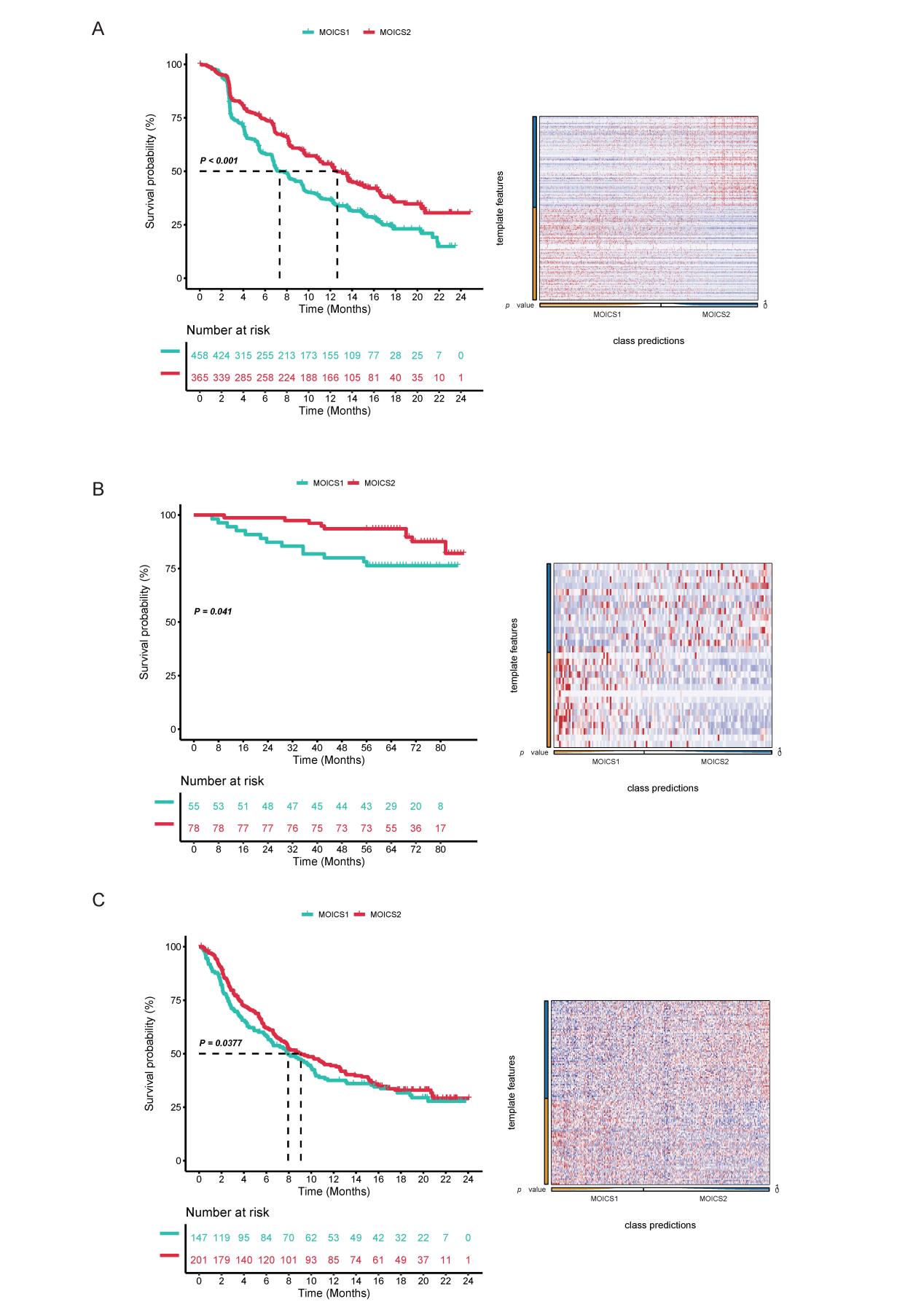


**Figure S5** Validation of clusters in out-house ccRCC cohorts. Consistency of reclusters based on the NTP algorithm and KM plot in Braun’s cohort (A); Qu’s cohort (B) and IMvigor210 dataset (C).


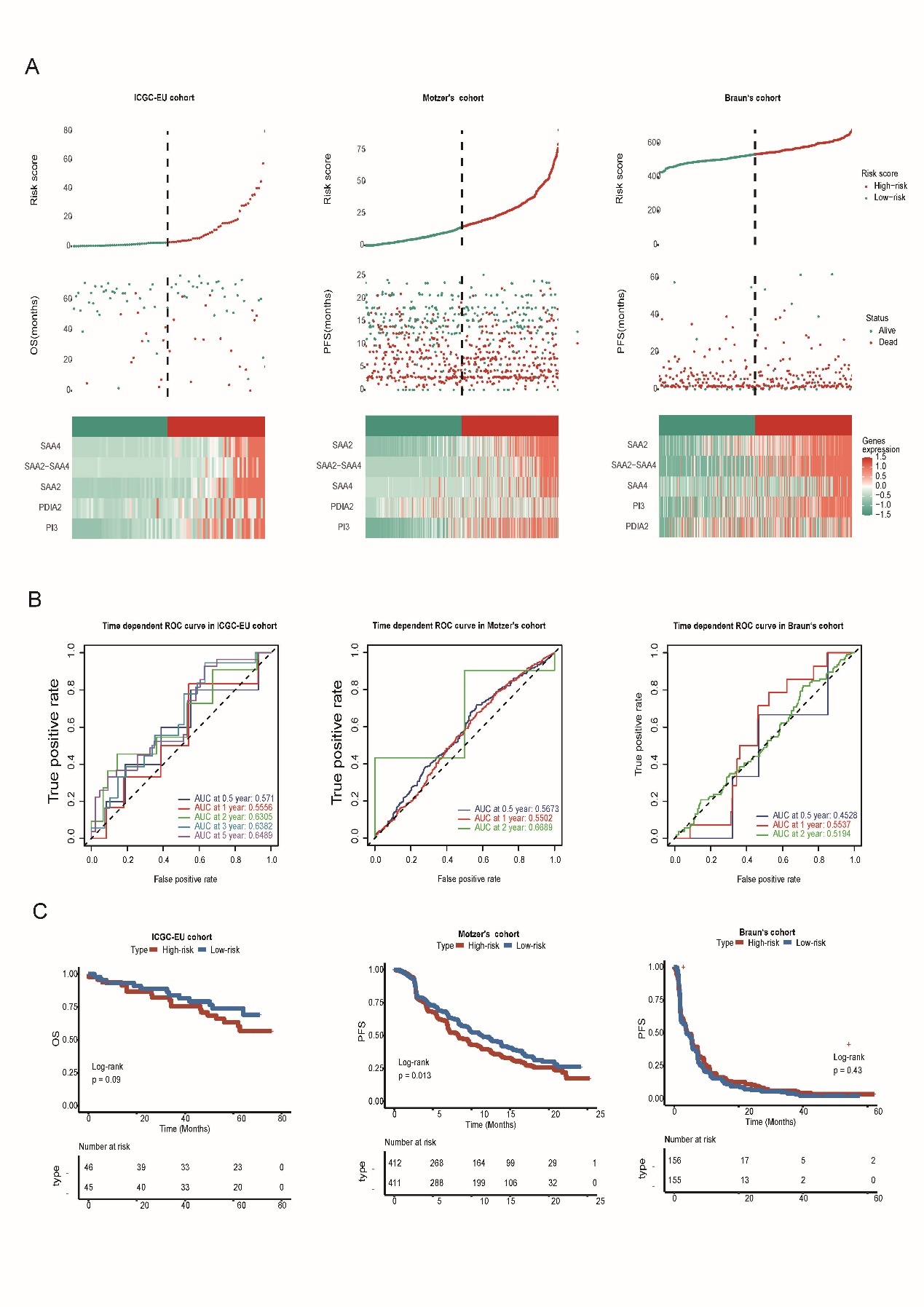


**Figure S6** Validation of risk score in three ccRCC cohorts. (A)Risk distribution plots, (B)receiver operating characteristic curves, and (C) Kaplan–Meier survival curves among ICGC-RU, Motzer’s, and Braun’s cohorts.


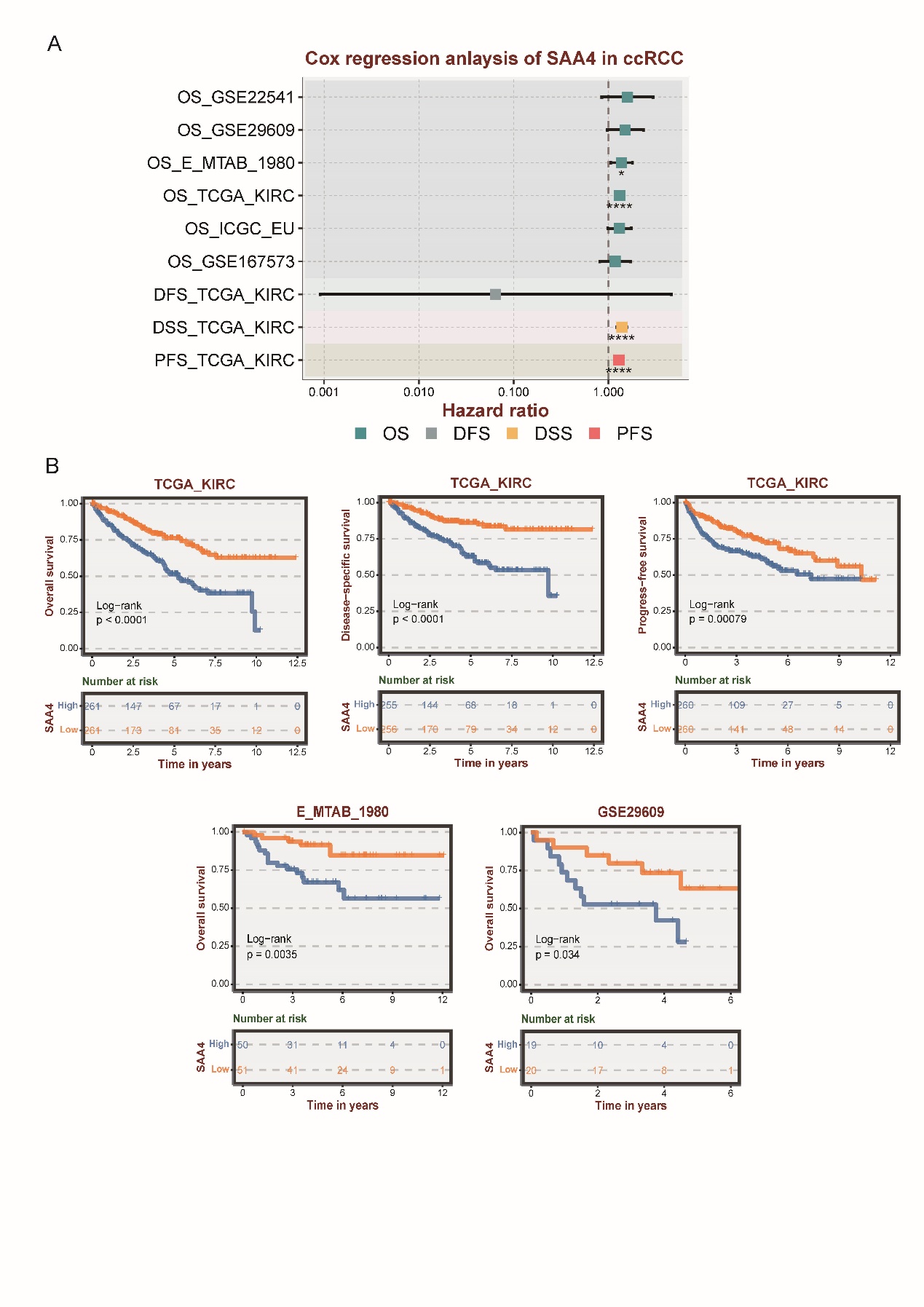


**Figure S7** Validation of the prognostic role of SAA4 across multi ccRCC datasets. (A) Cox regression analysis of SAA4 in multi ccRCC cohorts. (B) The survival analysis of different SAA4 expression groups based on median expression level in multi ccRCC cohorts.


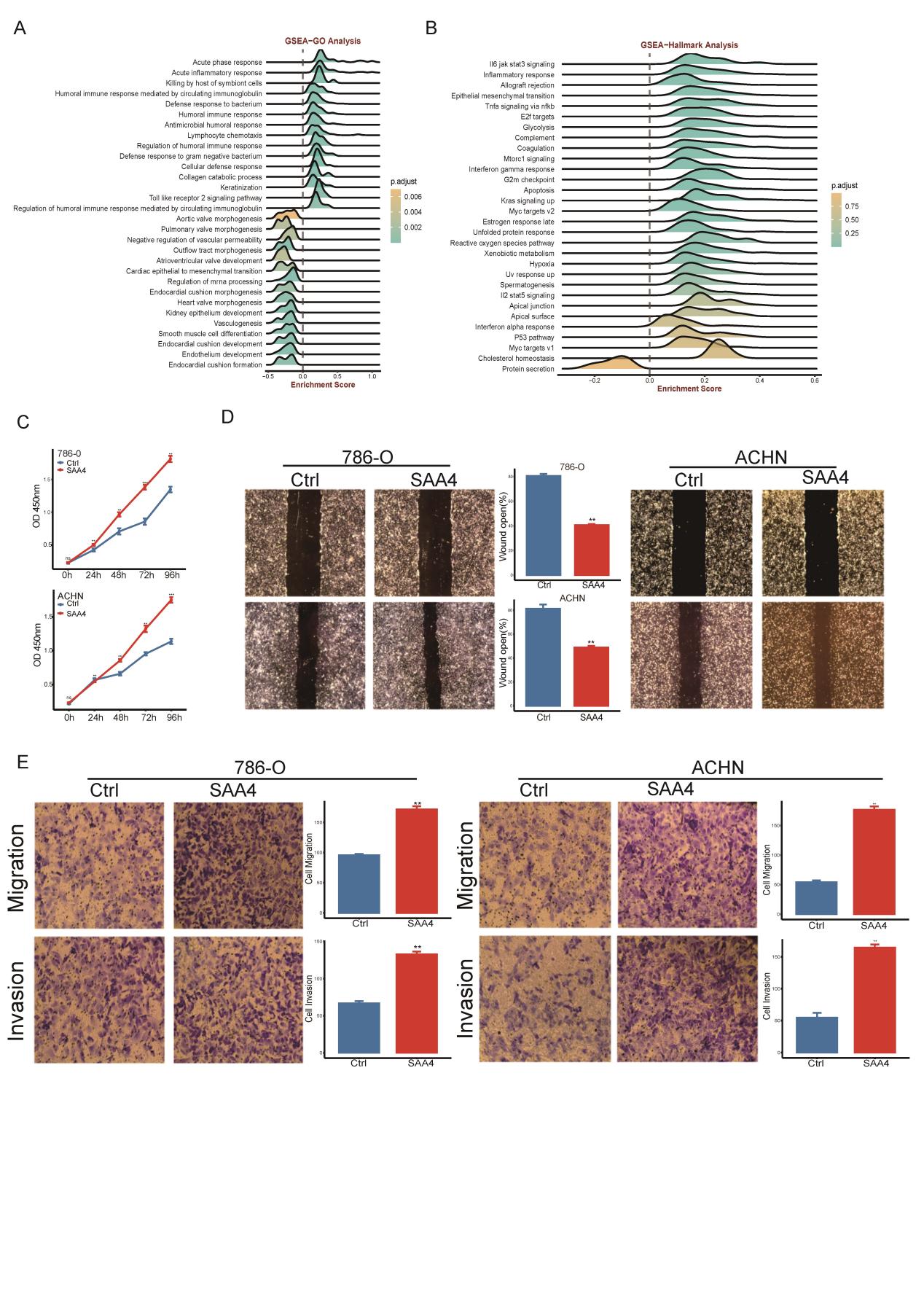


**Figure S8** (A) GO and (B) GSEA analysis of SAA4 in ccRCC based on association analysis. (C) Cell proliferation of 786-O and ACHN after being added with PBS or SAA4 in cell culture medium. (D) Wound healing assay and (E) number of cell invasion and migration of 786-O and ACHN cells treated with PBS or SAA4 in cell culture medium. ns, not significant; *, p < 0.05; **, p < 0.01; ***, p < 0.001; NC, negative control.


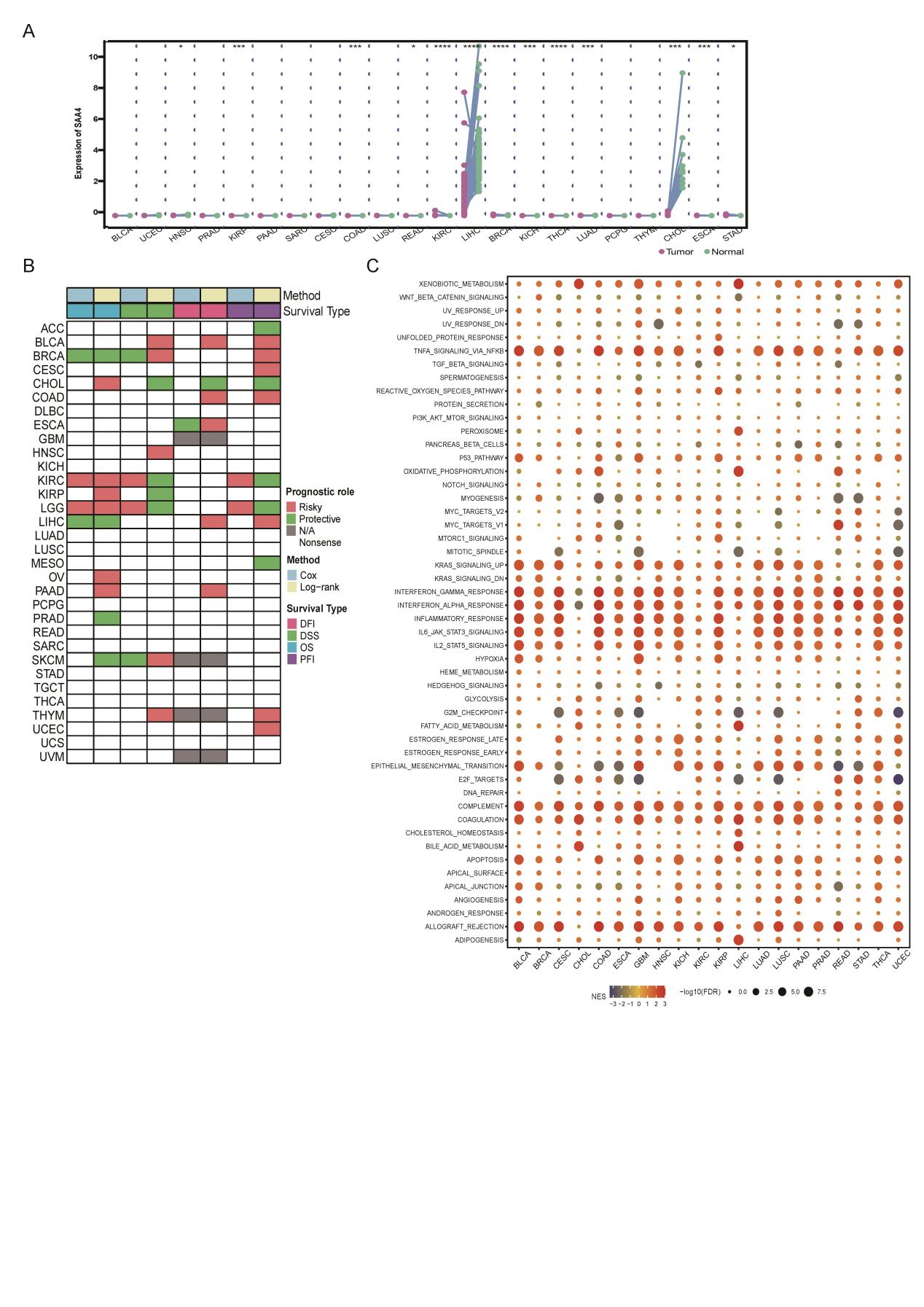


**Figure S9** (A) Different expression level of SAA4 across cancer types. (B) Prognostic impact including DFI, DSS, OS and PFI of SAA4 in multi cancer types. (C) Biological roles of SAA4 among cancer based on DEG extracted from SAA4 high and low expression groups.

**Supplementary Tables**

**Supplement Table 1** Baseline information of publica datasets enrolled in this work.

**Supplement Table 2** Different clinical characteristics between MOICS1 and MOICS2.

**Supplement Table 3** Biomarkers of MOICS1 and MOICS2.

**Supplement Table 4** Recurrently amplified and deleted regions between MOICS1 and MOICS2.

**Supplement Table 5** The potential target chemotherapy drugs for MOICS1 and MOICS2.
